# Supplementary material for: Comparison of parametric versus machine-learning multiple imputation in clinical trials with missing continuous outcomes
Source: BMC Med Res Methodol. 2026 Aug 3;26:232. doi: 10.1186/s12874-026-02962-6 (PMC13721589; doi:10.1186/s12874-026-02962-6)
Supplement: Supplementary file 1 [file 12874_2026_2962_MOESM1_ESM.pdf]

Supplementary File 1

1 Data Generating Mechanisms

1.1 Single Covariate Setting

We assume a baseline risk factor $x_i \sim N(0,1)$ and error term $e_i \sim N(0, 60^2)$ for all i. Outcomes are generated under each covariate-outcome relationship as follows:

- *Linear*:
$$y_i = 395 + 110x_i + 40\mathbb{I}(\text{arm}_i = 1) + e_i$$

- *Flattening-off*:
$$y_i = 700 - \exp(-x_i + 4) + 40 + 40\mathbb{I}(\text{arm}_i = 1) + e_i.$$

- *J-shaped*:
$$y_i = 200 + 100x_i + 60x_i^2 + 40\mathbb{I}(\text{arm}_i = 1) + e_i.$$

- *Two-tier*:
$$y_i = 180 + 470\mathbb{I}(x_i > 0) + 40\mathbb{I}(\text{arm}_i = 1) + e_i,$$

- *Full quadratic*
$$y_i = 100 + 104x_i^2 + 40\mathbb{I}(\text{arm}_i = 1) + e_i$$

- *Harmonic*:
$$y_i = 400 + 300\cos(0.9\pi x_i + 4) + 40\mathbb{I}(\text{arm}_i = 1) + e_i.$$

where $\mathbb{I}(\cdot)$ denotes the indicator function.

1.2 Covariate-treatment interaction

We assume a baseline risk factor $x_i \sim N(0,1)$ and error term $e_i \sim N(0, 60^2)$ for all i. Outcome under each interaction setting are generated as follows:

- *Small interaction*:
$$y_i = 395 + 80x_i + (20x_i + 40)\mathbb{I}(\text{arm}_i = 1) + e_i.$$

- *Large interaction*:

$$y_i = 500 + 20x_i + (90x_i - 65)\mathbb{I}(\text{arm}_i = 1) + e_i.$$

- *Different shapes*:

$$y_i = 450 + 100x_i + (100 + 100x_i - \exp(-(x_i - 3.5)))\mathbb{I}(\text{arm}_i = 1) + e_i.$$

- *Absent in one group*:

$$y_i = 13x_i^4\mathbb{I}(\text{arm}_i = 1) + e_i.$$

2 Implementation of Multiple Imputation approaches

We provide code snippets to illustrate the specific implementation of each MI approach. Across simulation studies, we perform MI separately by treatment arm. The data frame `df_arm0` has data for individuals assigned to treatment 0, with a variable `x` for the covariate and variable `y` for the outcome. We set `maxit` (the number of cycles) to 1, the outcome is the only variable with missing values.

We display code for MI-PMM, MI-norm, MI-RF, MI-CART and MI-SL. Note that for MI-SL, learners are first specified on line 5. The parameter `tuning` takes values of either 5, 10 or 20 in the single covariate setting.

```
1 imp_0 <- mice(df_arm0, m = 30, maxit=1,method="pmm")
2 imp_0 <- mice(df_arm0, m = 30, maxit=1,method="norm")
3 imp_0 <- mice(df_arm0, m = 30, maxit=1,method="rf", ntree=tuning)
4 imp_0 <- mice(df_arm0, m = 30, maxit=1,method="cart", minbucket=tuning)
5 SL.lib <- c("SL.mean", "SL.glm", "SL.gam", "SL.randomForest")
6 imp_0 <- mice(df_arm0, m=30, maxit=1, method="SuperLearner", SL.lib=SL.
    lib, kernel="gaussian", bw=c(0.25, 1, 5))
```

`R` code for the simulation studies are provided in the GitHub repository.

	Response	True Diff	Method	Sample Size	Missingness Mech	Tuning param	No. Failed reps
1	Two-tier	40.00	MI-PMM	50	MAR-X-30	none	40.00
2	Two-tier	40.00	MI-CART	50	MCAR30	10	40.00
3	Two-tier	40.00	MI-CART	50	MCAR30	20	40.00
4	Two-tier	40.00	MI-CART	50	MAR-Z-30	10	58.00
5	Two-tier	40.00	MI-CART	50	MAR-Z-30	20	58.00
6	Two-tier	40.00	MI-CART	50	MAR-X-30	5	39.00
7	Two-tier	40.00	MI-CART	50	MAR-X-30	10	53.00
8	Two-tier	40.00	MI-CART	50	MAR-X-30	20	53.00
9	Harmonic	0.00	MI-norm	50	MCAR30	none	45.00
10	Harmonic	0.00	MI-norm	50	MAR-Z-30	none	34.00
11	Harmonic	0.00	MI-norm	50	MAR-X-30	none	73.00
12	Harmonic	0.00	MI-PMM	50	MCAR30	none	46.00
13	Harmonic	0.00	MI-PMM	50	MAR-Z-30	none	44.00
14	Harmonic	0.00	MI-PMM	50	MAR-X-30	none	35.00
15	Harmonic	0.00	Complete Cases	50	MCAR30	none	33.00
16	Harmonic	0.00	Complete Cases	50	MAR-Z-30	none	43.00
17	Harmonic	0.00	Complete Cases	50	MAR-X-30	none	38.00
18	Harmonic	0.00	MI-RF	50	MCAR30	10	26.00
19	Harmonic	0.00	MI-RF	50	MCAR30	20	27.00
20	Harmonic	0.00	MI-RF	50	MAR-Z-30	20	33.00
21	Harmonic	0.00	MI-RF	50	MAR-X-30	5	29.00
22	Harmonic	0.00	MI-RF	50	MAR-X-30	10	29.00
23	Harmonic	0.00	MI-RF	50	MAR-X-30	20	31.00
24	Harmonic	0.00	MI-CART	50	MCAR30	5	27.00
25	Harmonic	0.00	MI-CART	50	MCAR30	10	32.00
26	Harmonic	0.00	MI-CART	50	MCAR30	20	32.00
27	Harmonic	0.00	MI-CART	50	MAR-Z-30	5	32.00
28	Harmonic	0.00	MI-CART	50	MAR-Z-30	10	46.00
29	Harmonic	0.00	MI-CART	50	MAR-Z-30	20	46.00
30	Harmonic	0.00	MI-CART	50	MAR-X-30	5	35.00
31	Harmonic	0.00	MI-CART	50	MAR-X-30	10	35.00
32	Harmonic	0.00	MI-CART	50	MAR-X-30	20	35.00
33	Harmonic	0.00	MI-SL	50	MAR-X-10	none	35.00
34	Harmonic	40.00	MI-norm	50	MCAR10	none	37.00
35	Harmonic	40.00	MI-norm	50	MCAR30	none	81.00
36	Harmonic	40.00	MI-norm	50	MAR-Z-10	none	44.00
37	Harmonic	40.00	MI-norm	50	MAR-Z-30	none	92.00
38	Harmonic	40.00	MI-norm	50	MAR-X-10	none	40.00
39	Harmonic	40.00	MI-norm	50	MAR-X-30	none	147.00
40	Harmonic	40.00	MI-PMM	50	MCAR10	none	34.00
41	Harmonic	40.00	MI-PMM	50	MCAR30	none	116.00
42	Harmonic	40.00	MI-PMM	50	MAR-Z-10	none	33.00
43	Harmonic	40.00	MI-PMM	50	MAR-Z-30	none	101.00
44	Harmonic	40.00	MI-PMM	50	MAR-X-10	none	35.00
45	Harmonic	40.00	MI-PMM	50	MAR-X-30	none	88.00
46	Harmonic	40.00	Complete Cases	50	MCAR10	none	28.00
47	Harmonic	40.00	Complete Cases	50	MCAR30	none	74.00
48	Harmonic	40.00	Complete Cases	50	MAR-Z-10	none	35.00
49	Harmonic	40.00	Complete Cases	50	MAR-Z-30	none	81.00
50	Harmonic	40.00	Complete Cases	50	MAR-X-10	none	27.00
51	Harmonic	40.00	Complete Cases	50	MAR-X-30	none	82.00
52	Harmonic	40.00	MI-RF	50	MCAR10	10	28.00
53	Harmonic	40.00	MI-RF	50	MCAR10	20	28.00
54	Harmonic	40.00	MI-RF	50	MCAR30	5	60.00
55	Harmonic	40.00	MI-RF	50	MCAR30	10	63.00
56	Harmonic	40.00	MI-RF	50	MCAR30	20	60.00
57	Harmonic	40.00	MI-RF	50	MAR-Z-10	5	26.00
58	Harmonic	40.00	MI-RF	50	MAR-Z-10	10	40.00
59	Harmonic	40.00	MI-RF	50	MAR-Z-30	5	79.00
60	Harmonic	40.00	MI-RF	50	MAR-Z-30	10	53.00
61	Harmonic	40.00	MI-RF	50	MAR-Z-30	20	61.00
62	Harmonic	40.00	MI-RF	50	MAR-X-10	5	28.00
63	Harmonic	40.00	MI-RF	50	MAR-X-10	20	29.00
64	Harmonic	40.00	MI-RF	50	MAR-X-30	5	75.00
65	Harmonic	40.00	MI-RF	50	MAR-X-30	10	63.00
66	Harmonic	40.00	MI-RF	50	MAR-X-30	20	67.00
67	Harmonic	40.00	MI-CART	50	MCAR10	10	35.00
68	Harmonic	40.00	MI-CART	50	MCAR10	20	35.00
69	Harmonic	40.00	MI-CART	50	MCAR30	5	69.00
70	Harmonic	40.00	MI-CART	50	MCAR30	10	67.00
71	Harmonic	40.00	MI-CART	50	MCAR30	20	67.00
72	Harmonic	40.00	MI-CART	50	MAR-Z-10	5	34.00
73	Harmonic	40.00	MI-CART	50	MAR-Z-10	10	31.00
74	Harmonic	40.00	MI-CART	50	MAR-Z-10	20	31.00
75	Harmonic	40.00	MI-CART	50	MAR-Z-30	5	75.00
76	Harmonic	40.00	MI-CART	50	MAR-Z-30	10	92.00
77	Harmonic	40.00	MI-CART	50	MAR-Z-30	20	92.00
78	Harmonic	40.00	MI-CART	50	MAR-X-10	5	36.00
79	Harmonic	40.00	MI-CART	50	MAR-X-10	10	33.00
80	Harmonic	40.00	MI-CART	50	MAR-X-10	20	33.00
81	Harmonic	40.00	MI-CART	50	MAR-X-30	5	91.00
82	Harmonic	40.00	MI-CART	50	MAR-X-30	10	75.00
83	Harmonic	40.00	MI-CART	50	MAR-X-30	20	75.00
84	Harmonic	40.00	MI-SL	50	MCAR10	none	30.00
85	Harmonic	40.00	MI-SL	50	MCAR30	none	42.00
86	Harmonic	40.00	MI-SL	50	MAR-Z-30	none	65.00
87	Harmonic	40.00	MI-SL	50	MAR-X-10	none	62.00

Table 1: Simulation settings where more than 25 repetitions (out of 5000) failed

Table 2: Time taken in seconds to run one repetition of a simulation under the single covariate setting. For brevity, we focus on the Linear and Full quadratic responses and the MCAR30 Missingness Mechanism.

	Method	Tuning	Sample size	Miss mech	True diff	Response	Time (s)
1	MI-norm	none	50	mcar30	40	Linear	0.253
2	MI-PMM	none	50	mcar30	40	Linear	0.255
3	Complete Cases	none	50	mcar30	40	Linear	0.004
4	MI-RF	5	50	mcar30	40	Linear	0.330
5	MI-RF	10	50	mcar30	40	Linear	0.355
6	MI-RF	20	50	mcar30	40	Linear	0.389
7	MI-CART	5	50	mcar30	40	Linear	0.370
8	MI-CART	10	50	mcar30	40	Linear	0.353
9	MI-CART	20	50	mcar30	40	Linear	0.347
10	MI-SL	none	50	mcar30	40	Linear	8.517
11	MI-norm	none	100	mcar30	40	Linear	0.254
12	MI-PMM	none	100	mcar30	40	Linear	0.262
13	Complete Cases	none	100	mcar30	40	Linear	0.004
14	MI-RF	5	100	mcar30	40	Linear	0.346
15	MI-RF	10	100	mcar30	40	Linear	0.370
16	MI-RF	20	100	mcar30	40	Linear	0.437
17	MI-CART	5	100	mcar30	40	Linear	0.373
18	MI-CART	10	100	mcar30	40	Linear	0.377
19	MI-CART	20	100	mcar30	40	Linear	0.352
20	MI-SL	none	100	mcar30	40	Linear	14.865
21	MI-norm	none	200	mcar30	40	Linear	0.260
22	MI-PMM	none	200	mcar30	40	Linear	0.263
23	Complete Cases	none	200	mcar30	40	Linear	0.004
24	MI-RF	5	200	mcar30	40	Linear	0.375
25	MI-RF	10	200	mcar30	40	Linear	0.424
26	MI-RF	20	200	mcar30	40	Linear	0.520
27	MI-CART	5	200	mcar30	40	Linear	0.392
28	MI-CART	10	200	mcar30	40	Linear	0.390
29	MI-CART	20	200	mcar30	40	Linear	0.382
30	MI-SL	none	200	mcar30	40	Linear	30.637
31	MI-norm	none	500	mcar30	40	Linear	0.270
32	MI-PMM	none	500	mcar30	40	Linear	0.281
33	Complete Cases	none	500	mcar30	40	Linear	0.003
34	MI-RF	5	500	mcar30	40	Linear	0.467
35	MI-RF	10	500	mcar30	40	Linear	0.569
36	MI-RF	20	500	mcar30	40	Linear	0.787
37	MI-CART	5	500	mcar30	40	Linear	0.431
38	MI-CART	10	500	mcar30	40	Linear	0.587
39	MI-CART	20	500	mcar30	40	Linear	0.428
40	MI-SL	none	500	mcar30	40	Linear	116.187
41	MI-norm	none	50	mcar30	40	Full quadratic	0.258
42	MI-PMM	none	50	mcar30	40	Full quadratic	0.257
43	Complete Cases	none	50	mcar30	40	Full quadratic	0.004
44	MI-RF	5	50	mcar30	40	Full quadratic	0.334
45	MI-RF	10	50	mcar30	40	Full quadratic	0.355
46	MI-RF	20	50	mcar30	40	Full quadratic	0.297
47	MI-CART	5	50	mcar30	40	Full quadratic	0.375
48	MI-CART	10	50	mcar30	40	Full quadratic	0.352
49	MI-CART	20	50	mcar30	40	Full quadratic	0.355
50	MI-SL	none	50	mcar30	40	Full quadratic	8.546
51	MI-norm	none	100	mcar30	40	Full quadratic	0.261
52	MI-PMM	none	100	mcar30	40	Full quadratic	0.261
53	Complete Cases	none	100	mcar30	40	Full quadratic	0.004
54	MI-RF	5	100	mcar30	40	Full quadratic	0.350
55	MI-RF	10	100	mcar30	40	Full quadratic	0.381
56	MI-RF	20	100	mcar30	40	Full quadratic	0.440
57	MI-CART	5	100	mcar30	40	Full quadratic	0.385
58	MI-CART	10	100	mcar30	40	Full quadratic	0.377
59	MI-CART	20	100	mcar30	40	Full quadratic	0.365
60	MI-SL	none	100	mcar30	40	Full quadratic	15.353
61	MI-norm	none	200	mcar30	40	Full quadratic	0.260
62	MI-PMM	none	200	mcar30	40	Full quadratic	0.269
63	Complete Cases	none	200	mcar30	40	Full quadratic	0.004
64	MI-RF	5	200	mcar30	40	Full quadratic	0.380
65	MI-RF	10	200	mcar30	40	Full quadratic	0.423
66	MI-RF	20	200	mcar30	40	Full quadratic	0.528
67	MI-CART	5	200	mcar30	40	Full quadratic	0.394
68	MI-CART	10	200	mcar30	40	Full quadratic	0.396
69	MI-CART	20	200	mcar30	40	Full quadratic	0.395
70	MI-SL	none	200	mcar30	40	Full quadratic	34.193
71	MI-norm	none	500	mcar30	40	Full quadratic	0.269
72	MI-PMM	none	500	mcar30	40	Full quadratic	0.281
73	Complete Cases	none	500	mcar30	40	Full quadratic	0.004
74	MI-RF	5	500	mcar30	40	Full quadratic	0.472
75	MI-RF	10	500	mcar30	40	Full quadratic	0.581
76	MI-RF	20	500	mcar30	40	Full quadratic	0.787
77	MI-CART	5	500	mcar30	40	Full quadratic	0.446
78	MI-CART	10	500	mcar30	40	Full quadratic	0.432
79	MI-CART	20	500	mcar30	40	Full quadratic	0.431
80	MI-SL	none	500	mcar30	40	Full quadratic	122.847
