# Supplementary material for: Comparison of parametric versus machine-learning multiple imputation in clinical trials with missing continuous outcomes
Source: BMC Med Res Methodol. 2026 Aug 3;26:232. doi: 10.1186/s12874-026-02962-6 (PMC13721589; doi:10.1186/s12874-026-02962-6)
Supplement: Supplementary file 2 [file 12874_2026_2962_MOESM2_ESM.pdf]

Supplementary File 2

In this supplementary file, we display additional simulation results.

1 Single covariate setting without interaction

1.1 Sample size 50

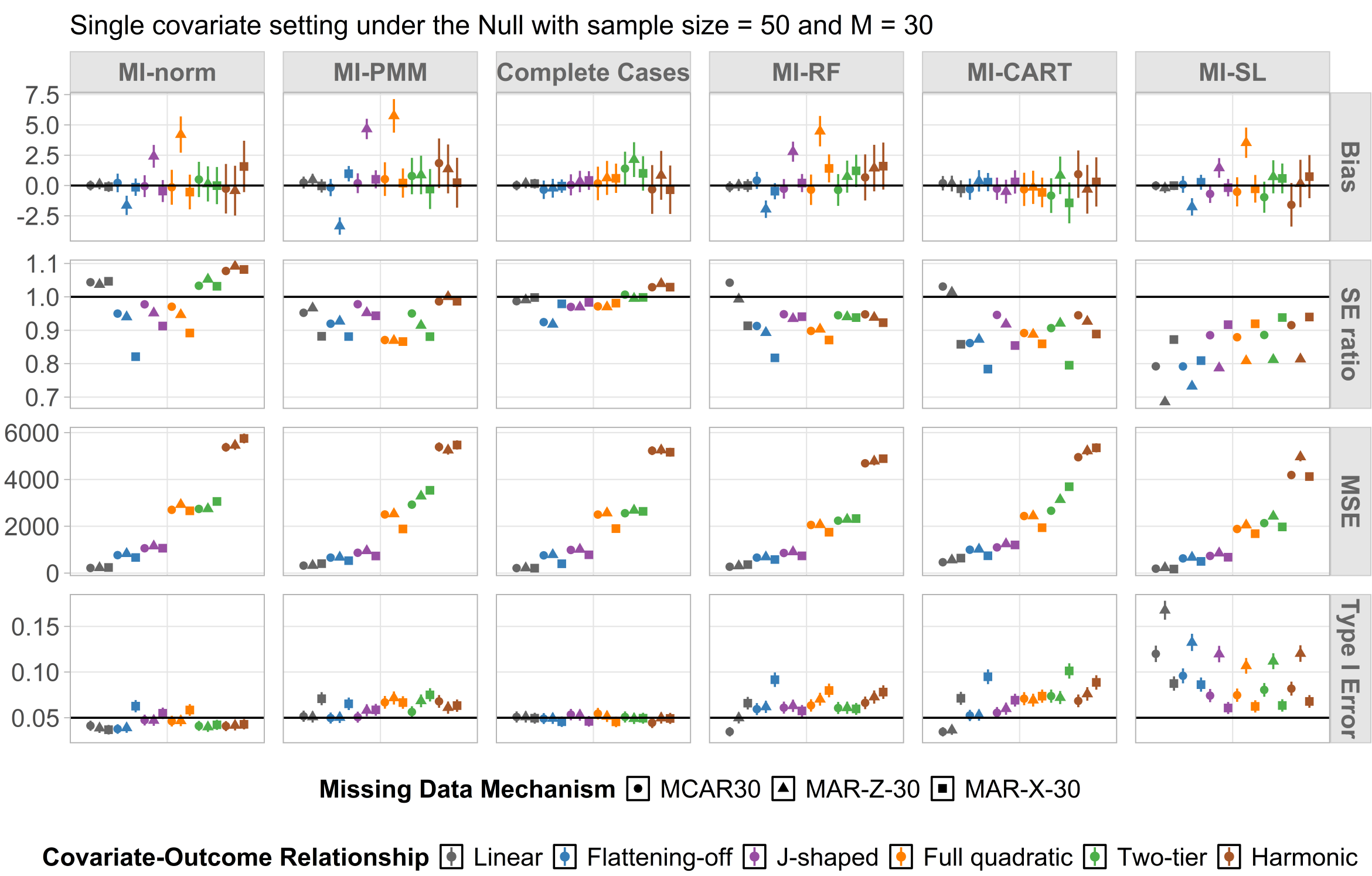

Figure 1: Simulation results for the single covariate, no interaction setting for sample size 50 when the true treatment effect is 0. The number of imputations is 30. Performance of missing data methods in terms of bias, ratio of model-based versus empirical standard error and power are provided across seven different missing data mechanisms. Note: for RF, the number of trees is set to 5 and for CART, minbucket is set to 5. Estimates are indicated with $\pm 1.96\times$ Monte Carlo error bars (but are often too small to be seen due to the scale of the plots).

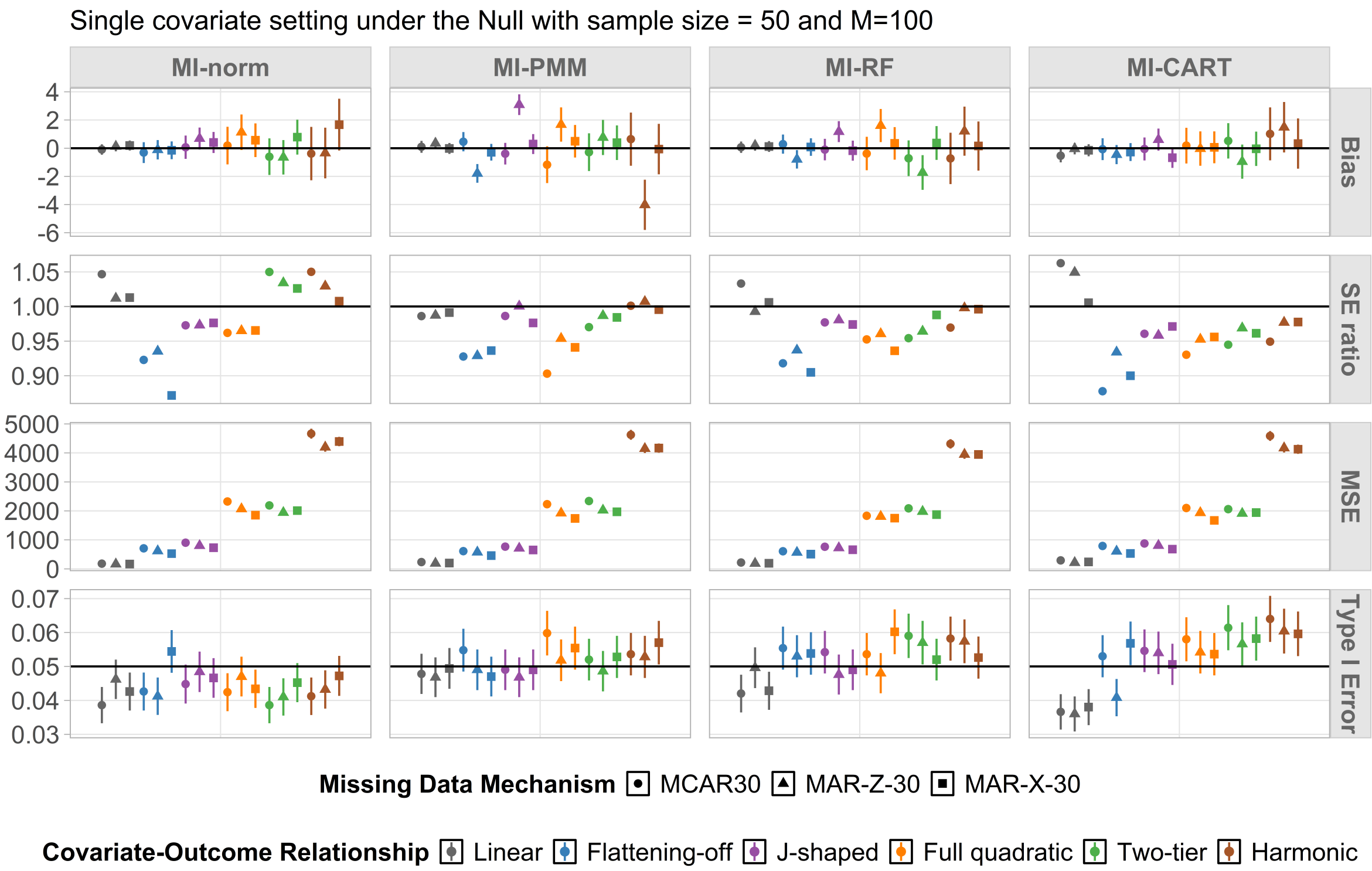

Figure 2: Simulation results for the single covariate, no interaction setting for sample size 50 when the true treatment effect is 0. The number of imputations is 100. Performance of missing data methods in terms of bias, ratio of model-based versus empirical standard error and power are provided across seven different missing data mechanisms. Note: for RF, the number of trees is set to 5 and for CART, minbucket is set to 5. Estimates are indicated with $\pm 1.96\times$ Monte Carlo error bars (but are often too small to be seen due to the scale of the plots).

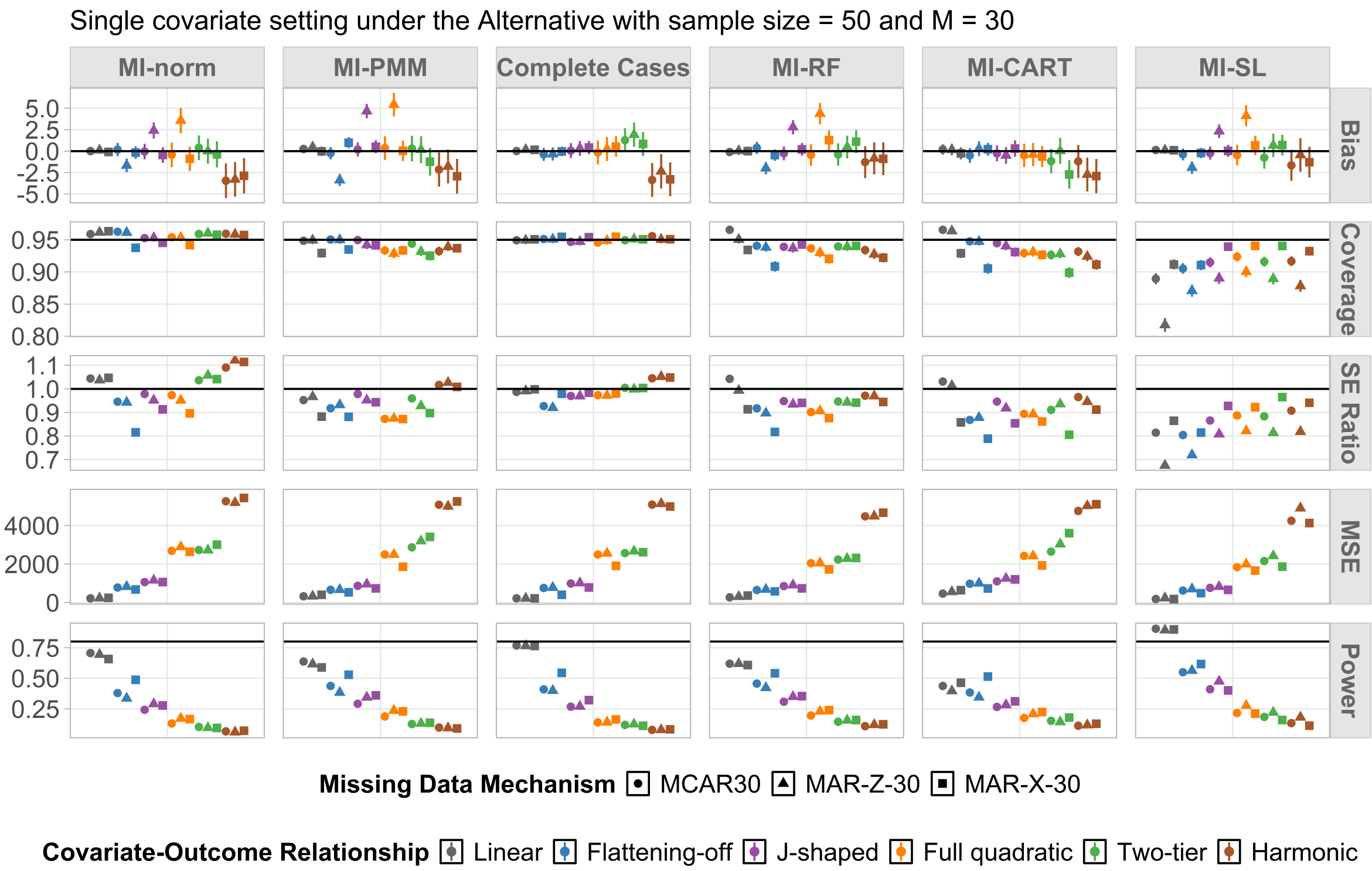

Figure 3: Simulation results for the single covariate, no interaction setting for sample size 50 when the true treatment effect is 40. The number of imputations is 30. Performance of missing data methods in terms of bias, ratio of model-based versus empirical standard error and power are provided across seven different missing data mechanisms. Note: for RF, the number of trees is set to 5 and for CART, minbucket is set to 5. Estimates are indicated with $\pm 1.96\times$ Monte Carlo error bars (but are often too small to be seen due to the scale of the plots).

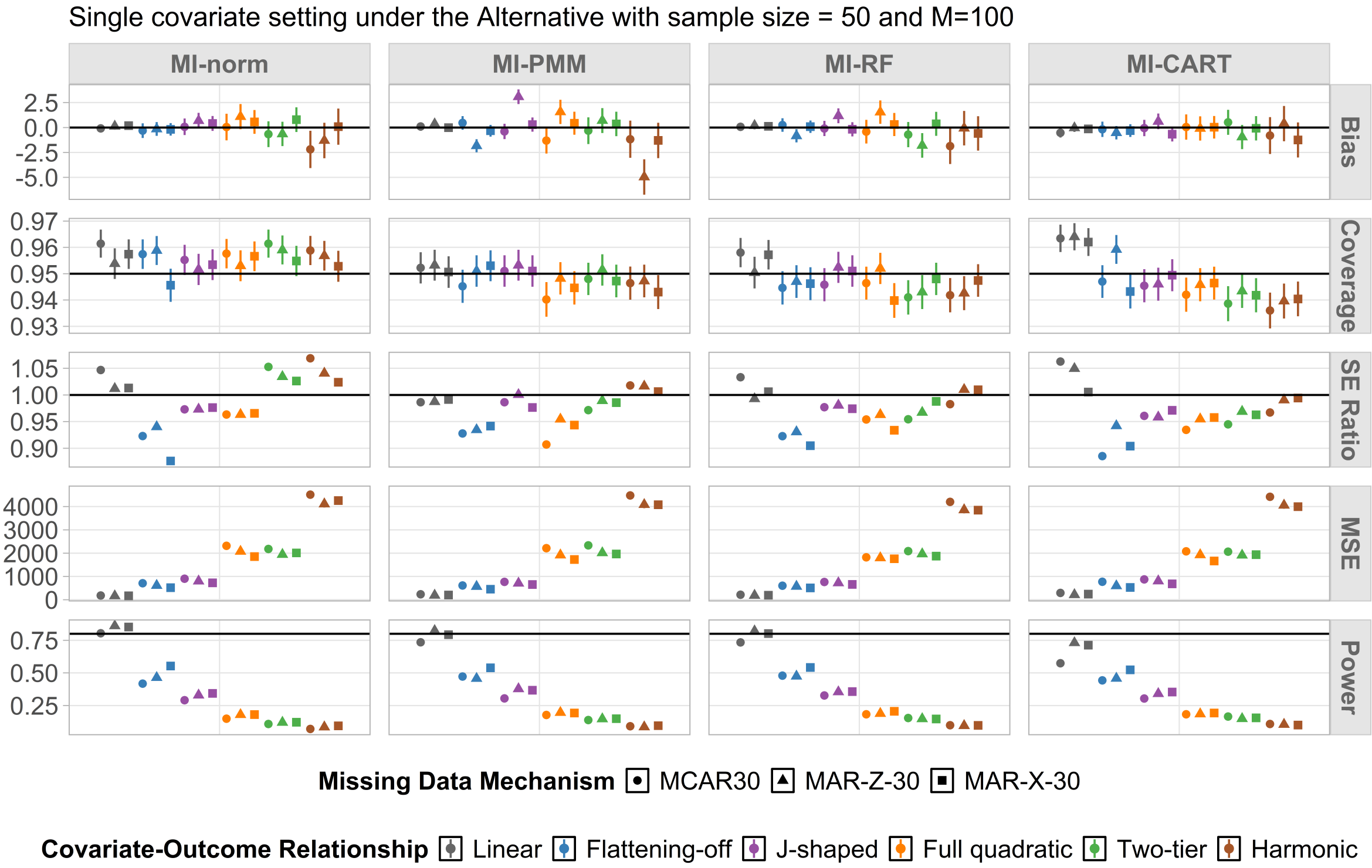

Figure 4: Simulation results for the single covariate, no interaction setting for sample size 50 when the true treatment effect is 40. The number of imputations is 100. Performance of missing data methods in terms of bias, ratio of model-based versus empirical standard error and power are provided across seven different missing data mechanisms. Note: for RF, the number of trees is set to 5 and for CART, minbucket is set to 5. Estimates are indicated with $\pm 1.96\times$ Monte Carlo error bars (but are often too small to be seen due to the scale of the plots).

1.2 Sample size 100

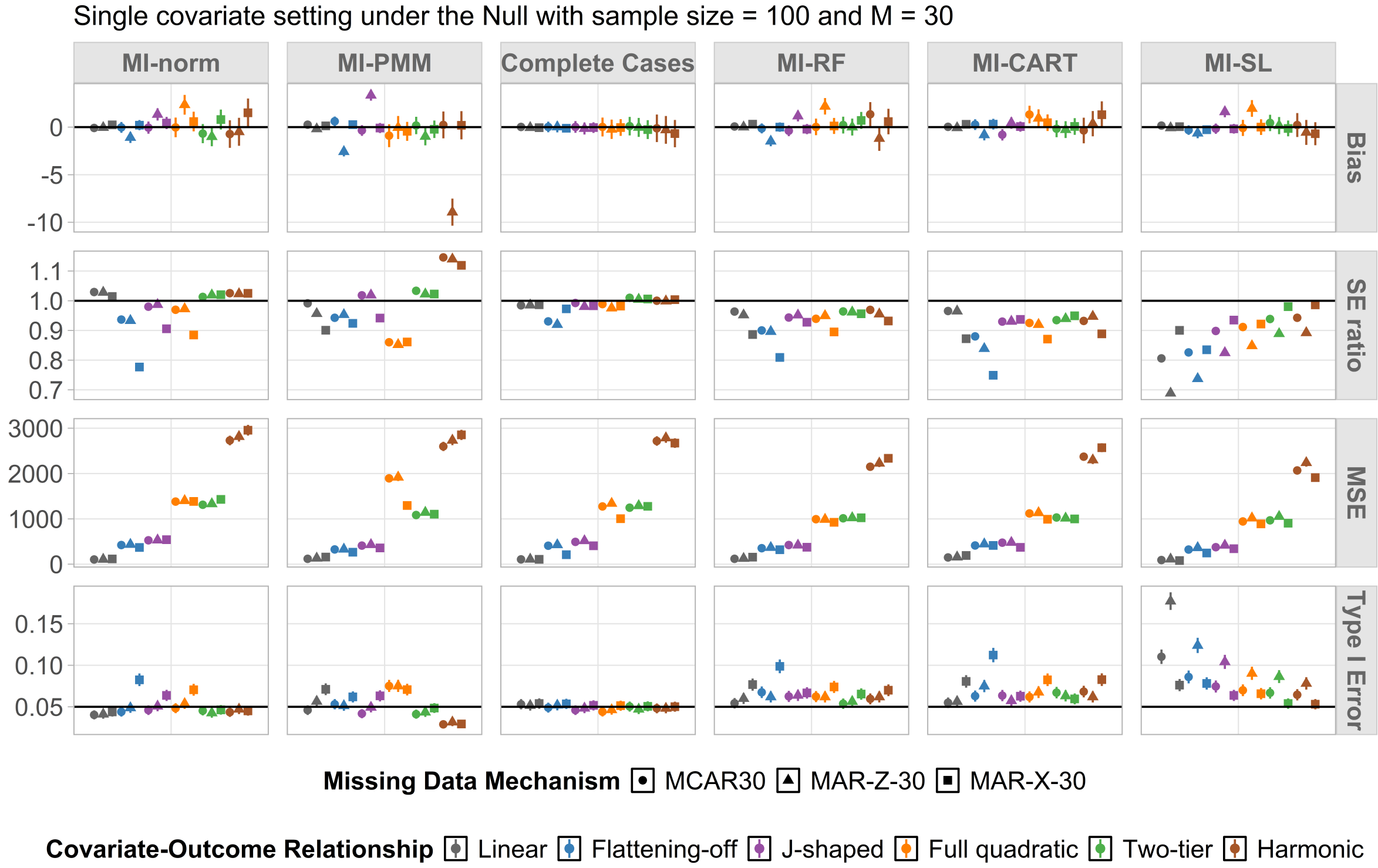

Figure 5: Simulation results for the single covariate, no interaction setting for sample size 100 when the true treatment effect is 0. The number of imputations is 30. Performance of missing data methods in terms of bias, ratio of model-based versus empirical standard error and power are provided across seven different missing data mechanisms. Note: for RF, the number of trees is set to 5 and for CART, minbucket is set to 5. Estimates are indicated with $\pm 1.96\times$ Monte Carlo error bars (but are often too small to be seen due to the scale of the plots).

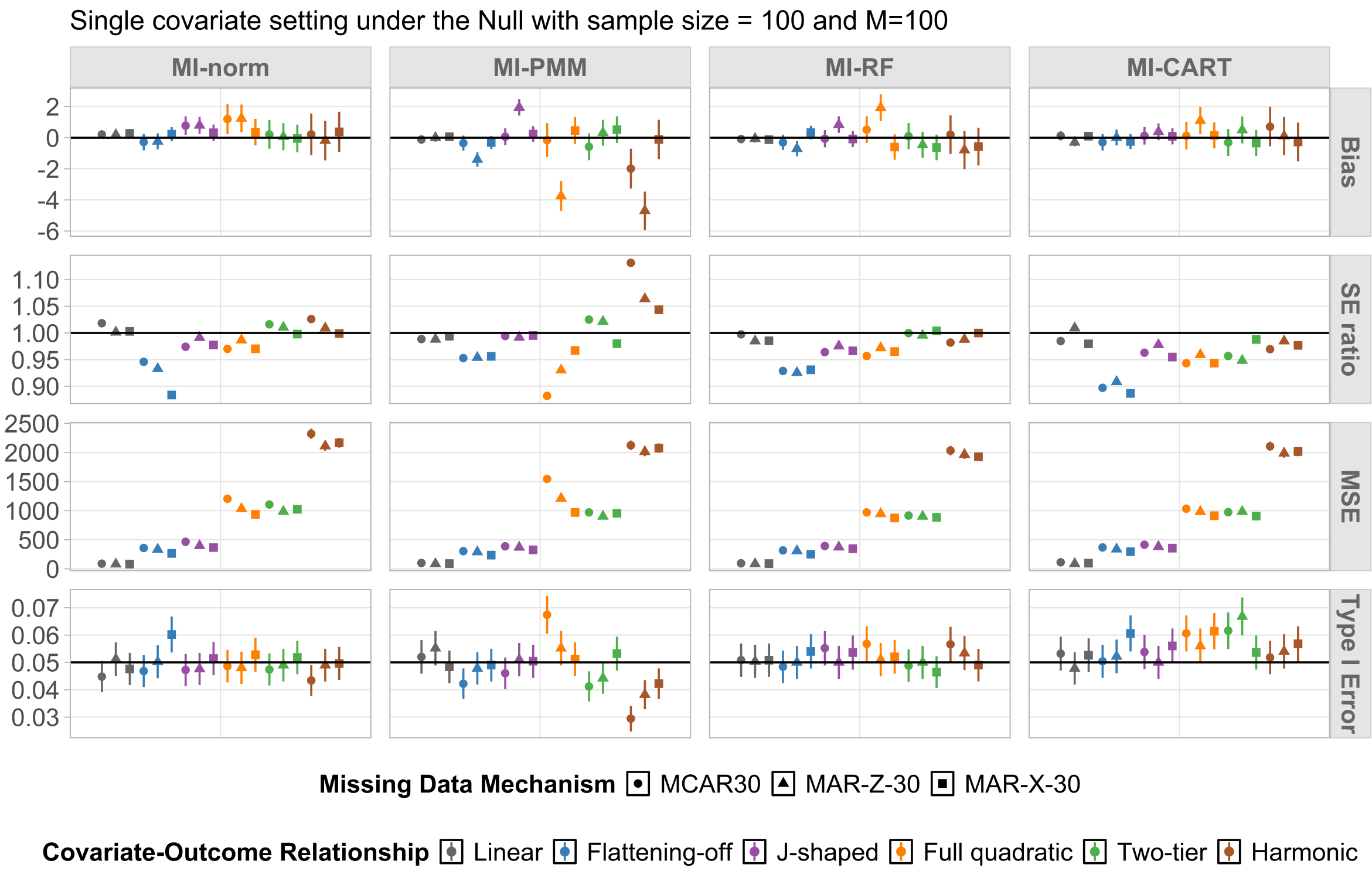

Figure 6: Simulation results for the single covariate, no interaction setting for sample size 100 when the true treatment effect is 0. The number of imputations is 100. Performance of missing data methods in terms of bias, ratio of model-based versus empirical standard error and power are provided across seven different missing data mechanisms. Note: for RF, the number of trees is set to 5 and for CART, minbucket is set to 5. Estimates are indicated with $\pm 1.96\times$ Monte Carlo error bars (but are often too small to be seen due to the scale of the plots).

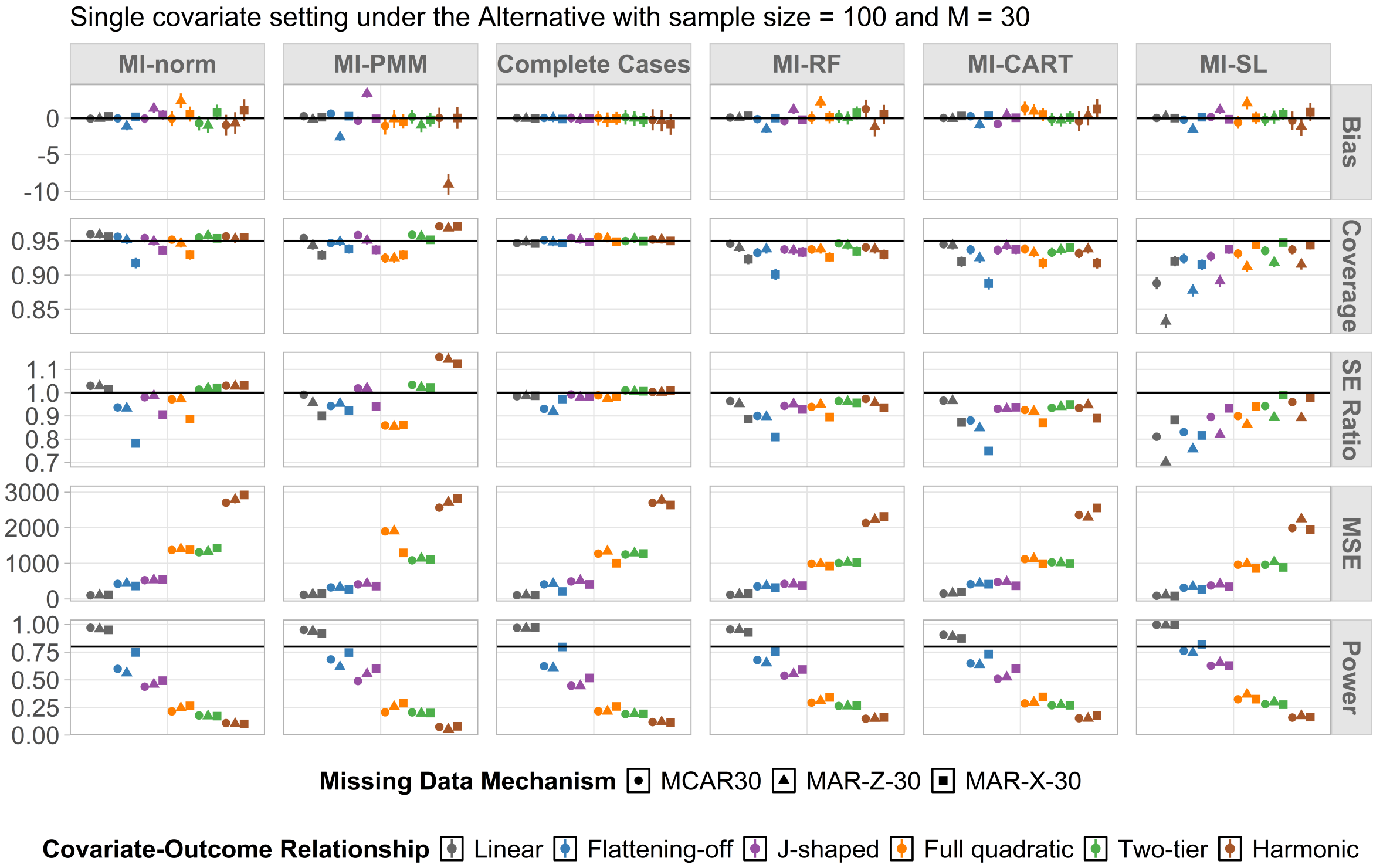

Figure 7: Simulation results for the single covariate, no interaction setting for sample size 100 when the true treatment effect is 40. The number of imputations is 30. Performance of missing data methods in terms of bias, ratio of model-based versus empirical standard error and power are provided across seven different missing data mechanisms. Note: for RF, the number of trees is set to 5 and for CART, minbucket is set to 5. Estimates are indicated with ±1.96× Monte Carlo error bars (but are often too small to be seen due to the scale of the plots).

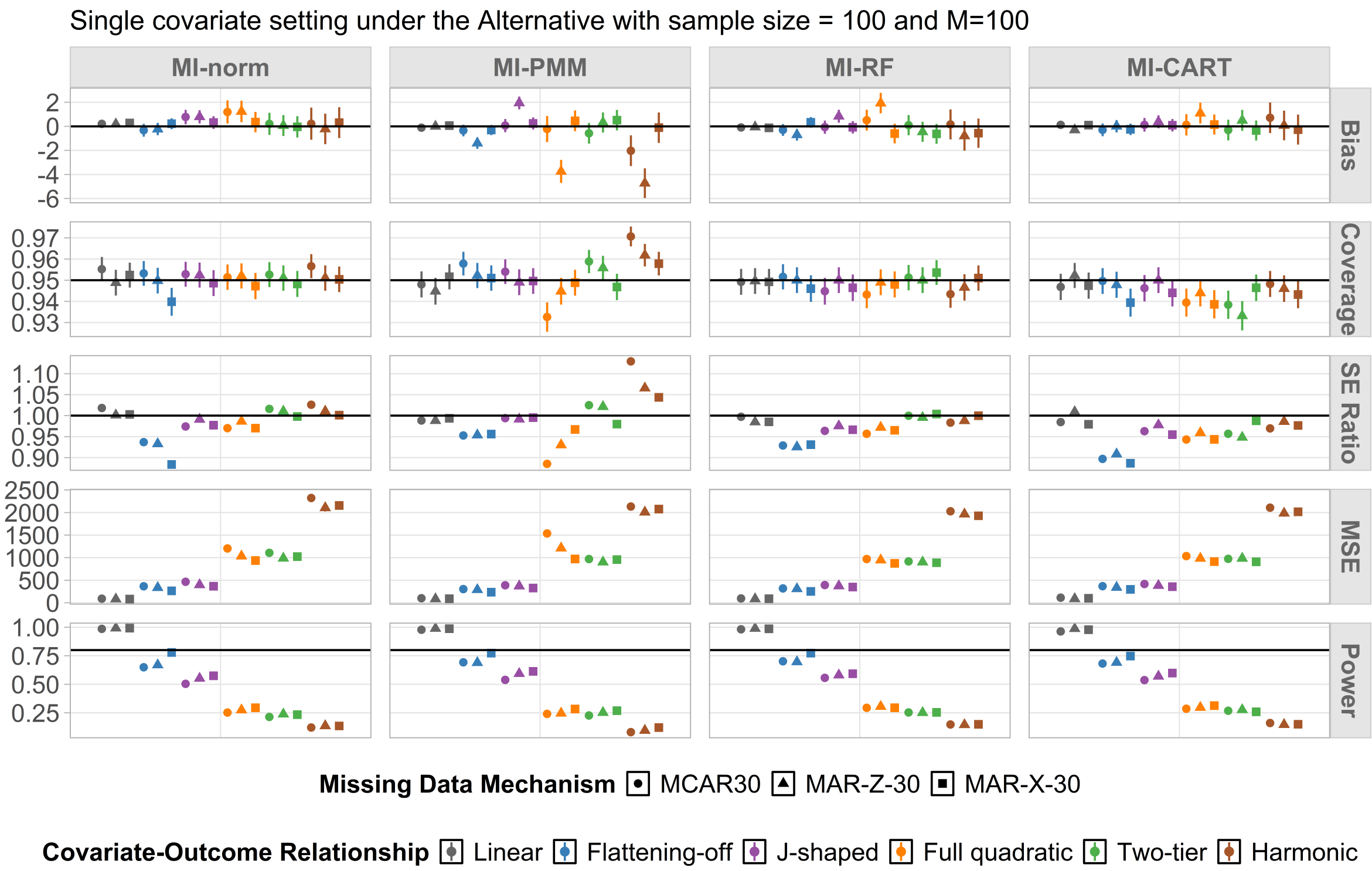

Figure 8: Simulation results for the single covariate, no interaction setting for sample size 100 when the true treatment effect is 40. The number of imputations is 100. Performance of missing data methods in terms of bias, ratio of model-based versus empirical standard error and power are provided across seven different missing data mechanisms. Note: for RF, the number of trees is set to 5 and for CART, minbucket is set to 5. Estimates are indicated with $\pm 1.96\times$ Monte Carlo error bars (but are often too small to be seen due to the scale of the plots).

1.3 Sample size 200

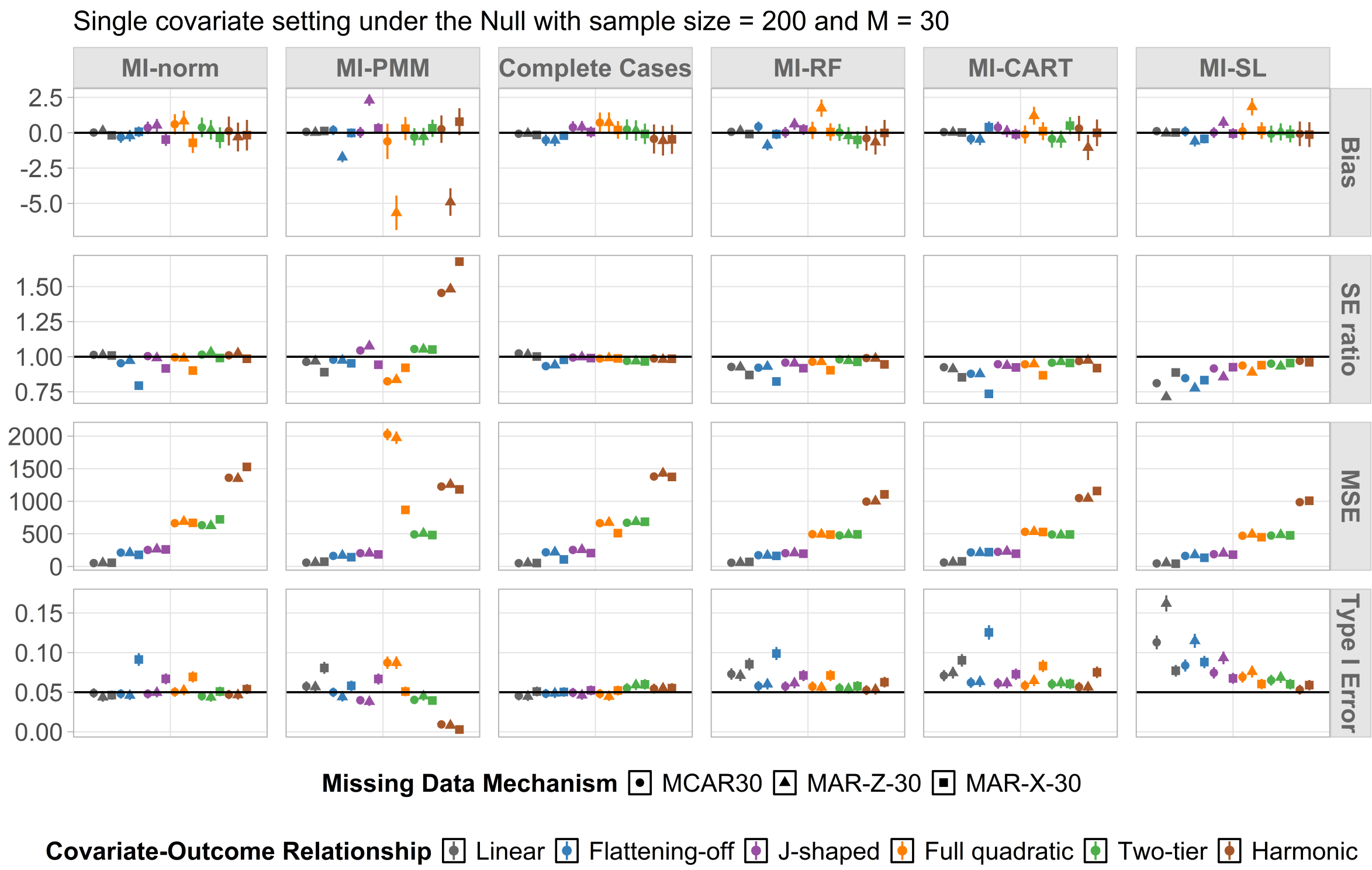

Figure 9: Simulation results for the single covariate, no interaction setting for sample size 200 when the true treatment effect is 0. The number of imputations is 30. Performance of missing data methods in terms of bias, ratio of model-based versus empirical standard error and power are provided across seven different missing data mechanisms. Note: for RF, the number of trees is set to 5 and for CART, minbucket is set to 5. Estimates are indicated with $\pm 1.96\times$ Monte Carlo error bars (but are often too small to be seen due to the scale of the plots).

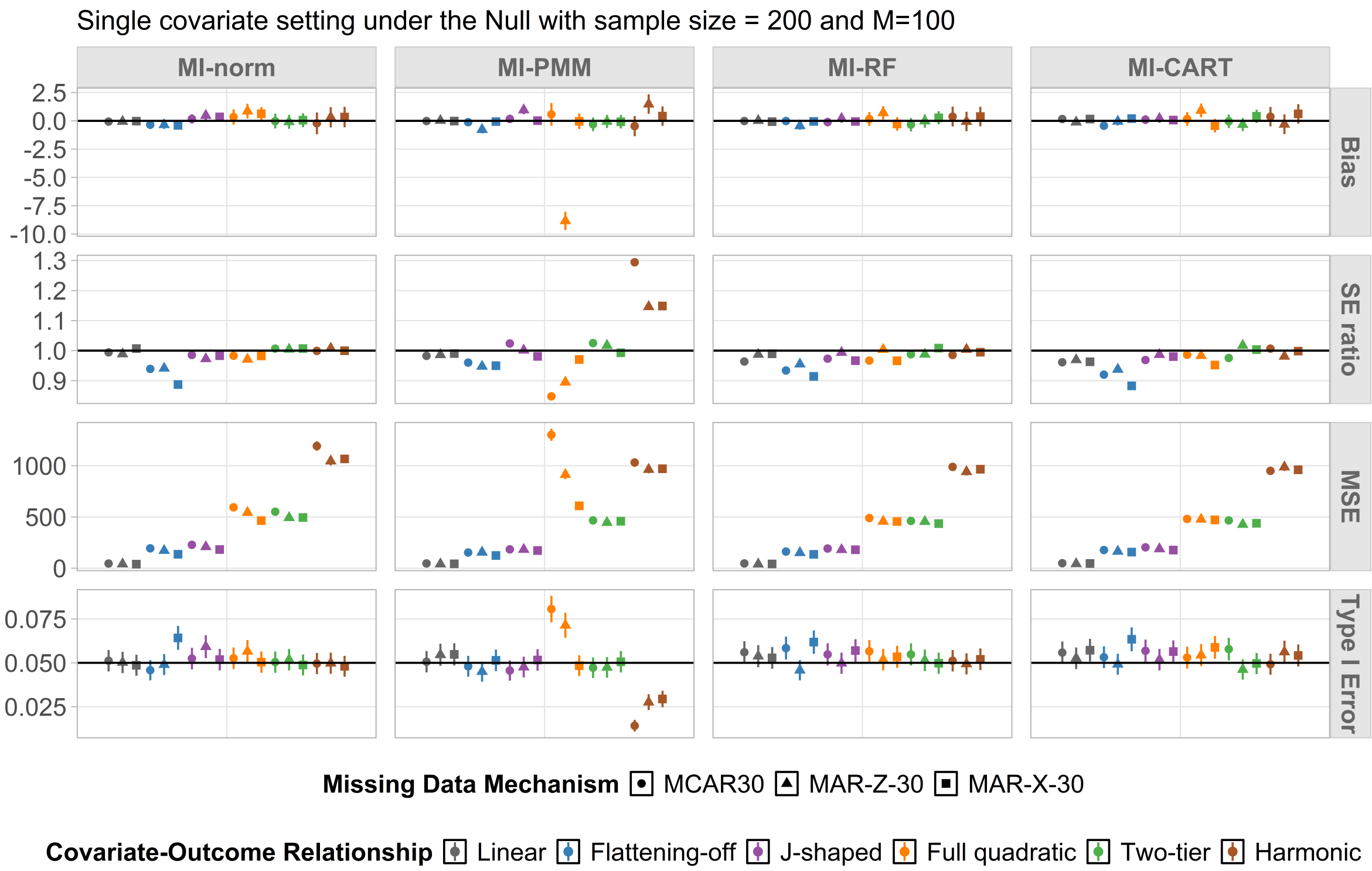

Figure 10: Simulation results for the single covariate, no interaction setting for sample size 200 when the true treatment effect is 0. The number of imputations is 100. Performance of missing data methods in terms of bias, ratio of model-based versus empirical standard error and power are provided across seven different missing data mechanisms. Note: for RF, the number of trees is set to 5 and for CART, minbucket is set to 5. Estimates are indicated with $\pm 1.96\times$ Monte Carlo error bars (but are often too small to be seen due to the scale of the plots).

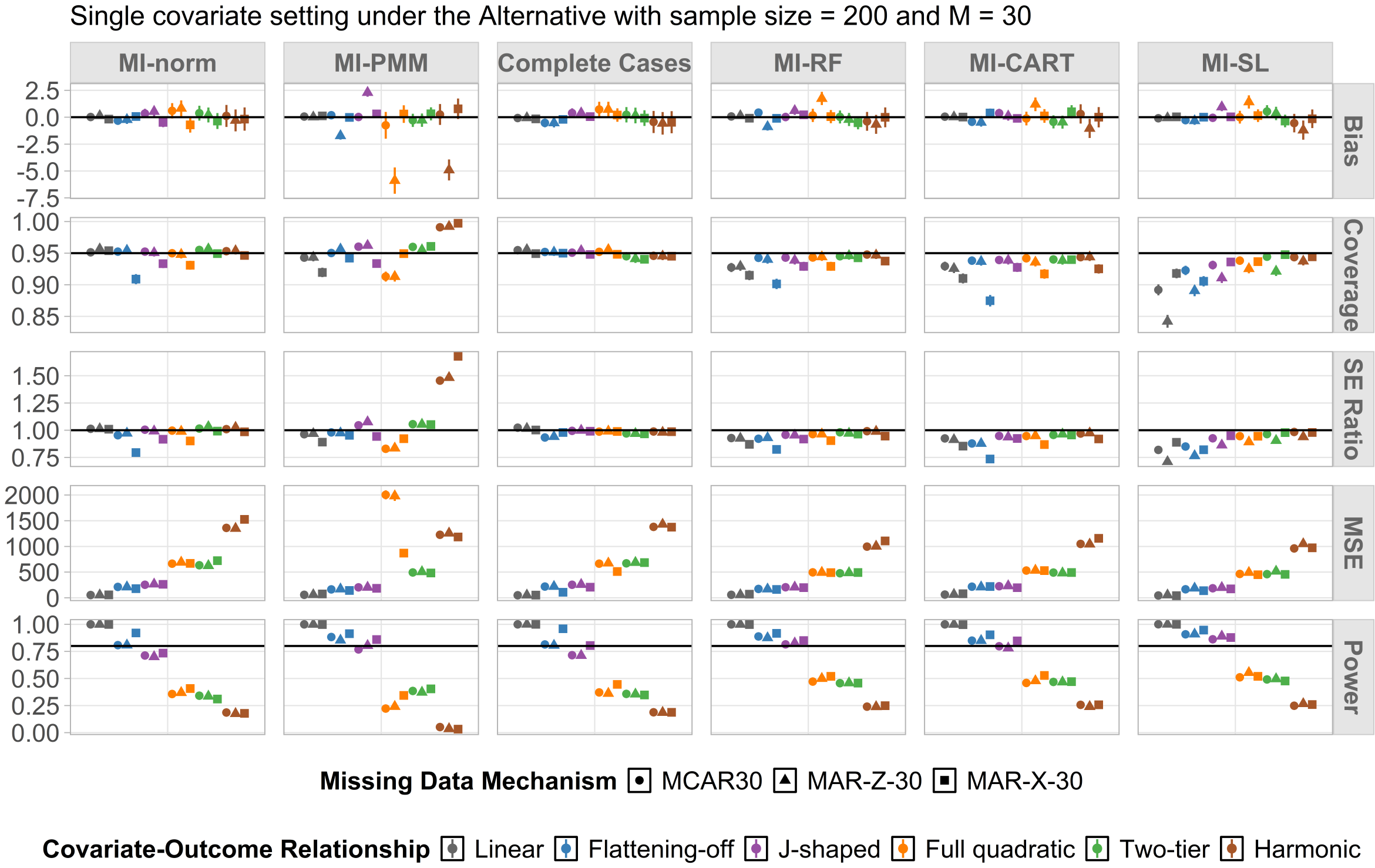

Figure 11: Simulation results for the single covariate, no interaction setting for sample size 200 when the true treatment effect is 40. The number of imputations is 30. Performance of missing data methods in terms of bias, ratio of model-based versus empirical standard error and power are provided across seven different missing data mechanisms. Note: for RF, the number of trees is set to 5 and for CART, minbucket is set to 5. Estimates are indicated with $\pm 1.96\times$ Monte Carlo error bars (but are often too small to be seen due to the scale of the plots).

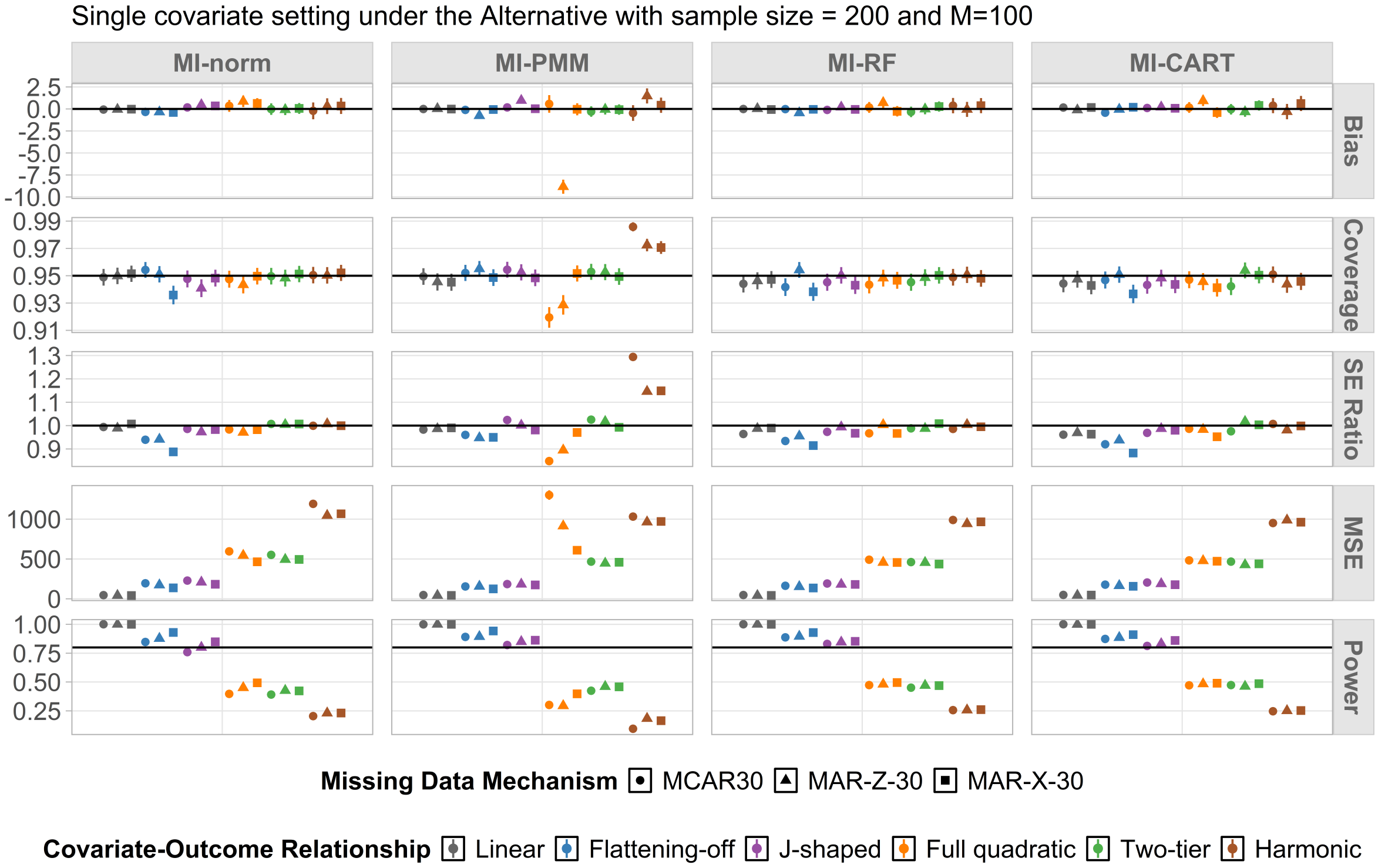

Figure 12: Simulation results for the single covariate, no interaction setting for sample size 200 when the true treatment effect is 40. The number of imputations is 100. Performance of missing data methods in terms of bias, ratio of model-based versus empirical standard error and power are provided across seven different missing data mechanisms. Note: for RF, the number of trees is set to 5 and for CART, minbucket is set to 5. Estimates are indicated with ±1.96× Monte Carlo error bars (but are often too small to be seen due to the scale of the plots).

1.4 Sample size 500

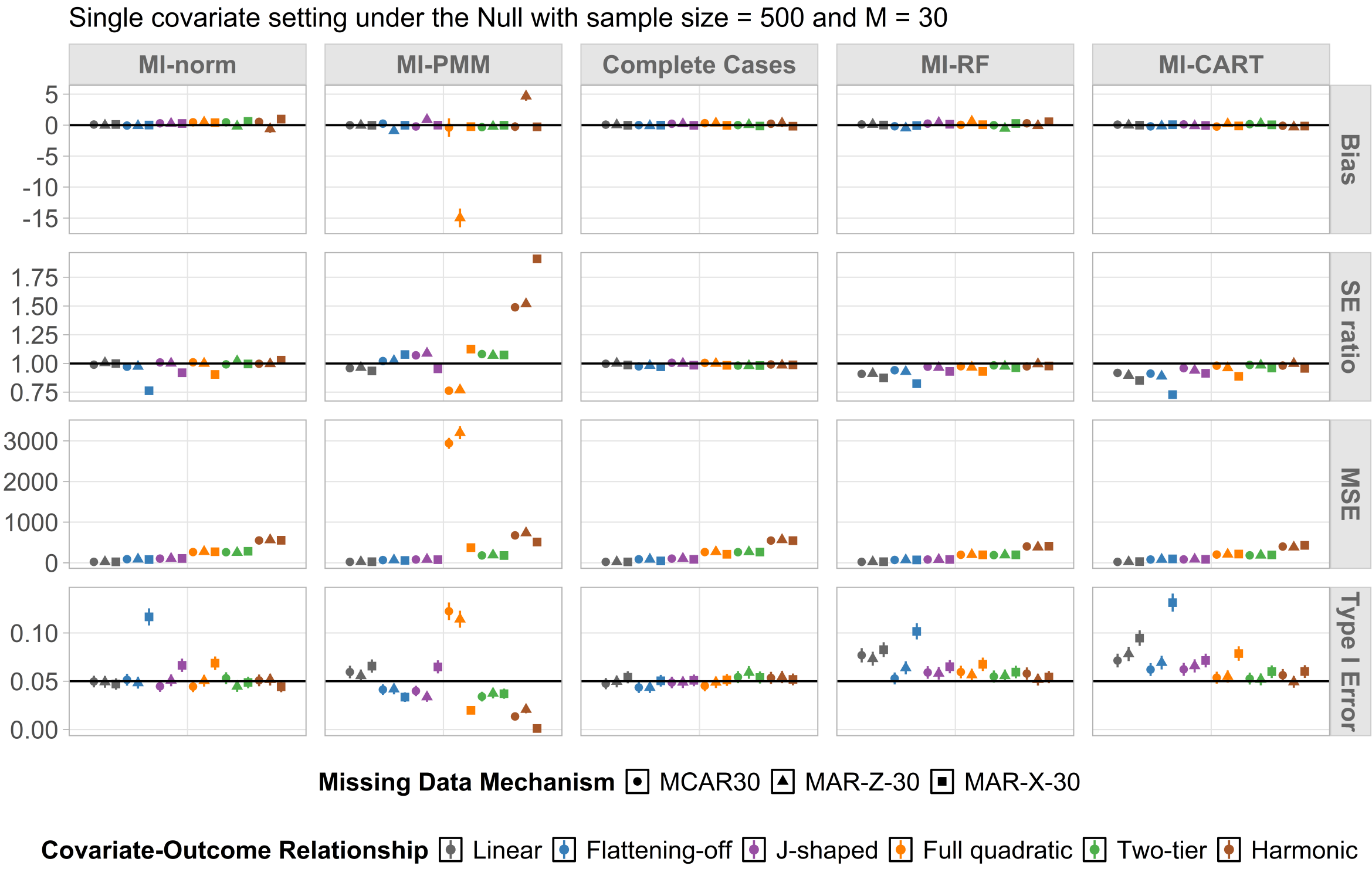

Figure 13: Simulation results for the single covariate, no interaction setting for sample size 500 when the true treatment effect is 0. The number of imputations is 30. Performance of missing data methods in terms of bias, ratio of model-based versus empirical standard error and power are provided across seven different missing data mechanisms. Note: for RF, the number of trees is set to 5 and for CART, minbucket is set to 5. Estimates are indicated with $\pm 1.96\times$ Monte Carlo error bars (but are often too small to be seen due to the scale of the plots).

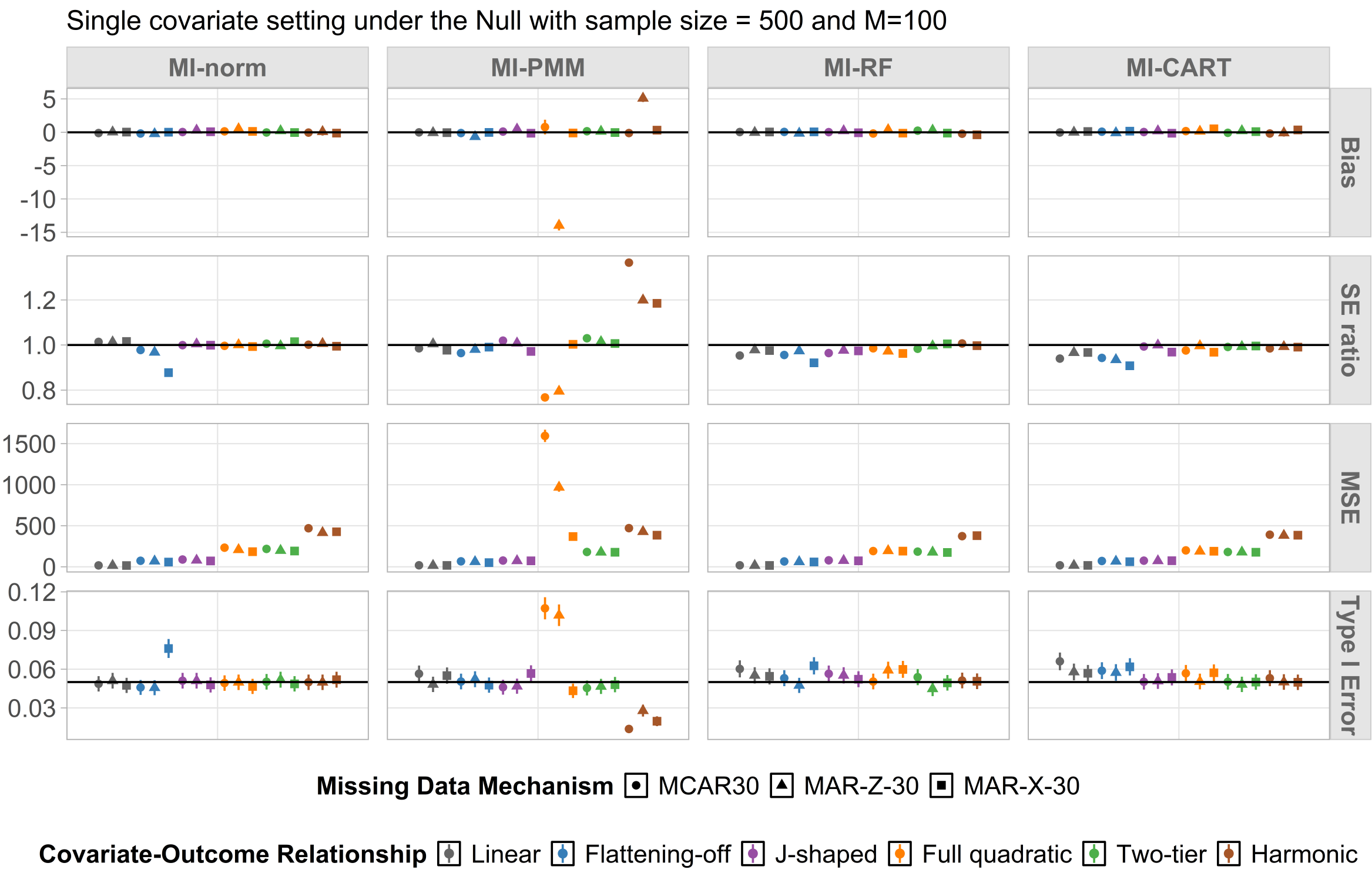

Figure 14: Simulation results for the single covariate, no interaction setting for sample size 500 when the true treatment effect is 0. The number of imputations is 100. Performance of missing data methods in terms of bias, ratio of model-based versus empirical standard error and power are provided across seven different missing data mechanisms. Note: for RF, the number of trees is set to 5 and for CART, minbucket is set to 5. Estimates are indicated with $\pm 1.96\times$ Monte Carlo error bars (but are often too small to be seen due to the scale of the plots).

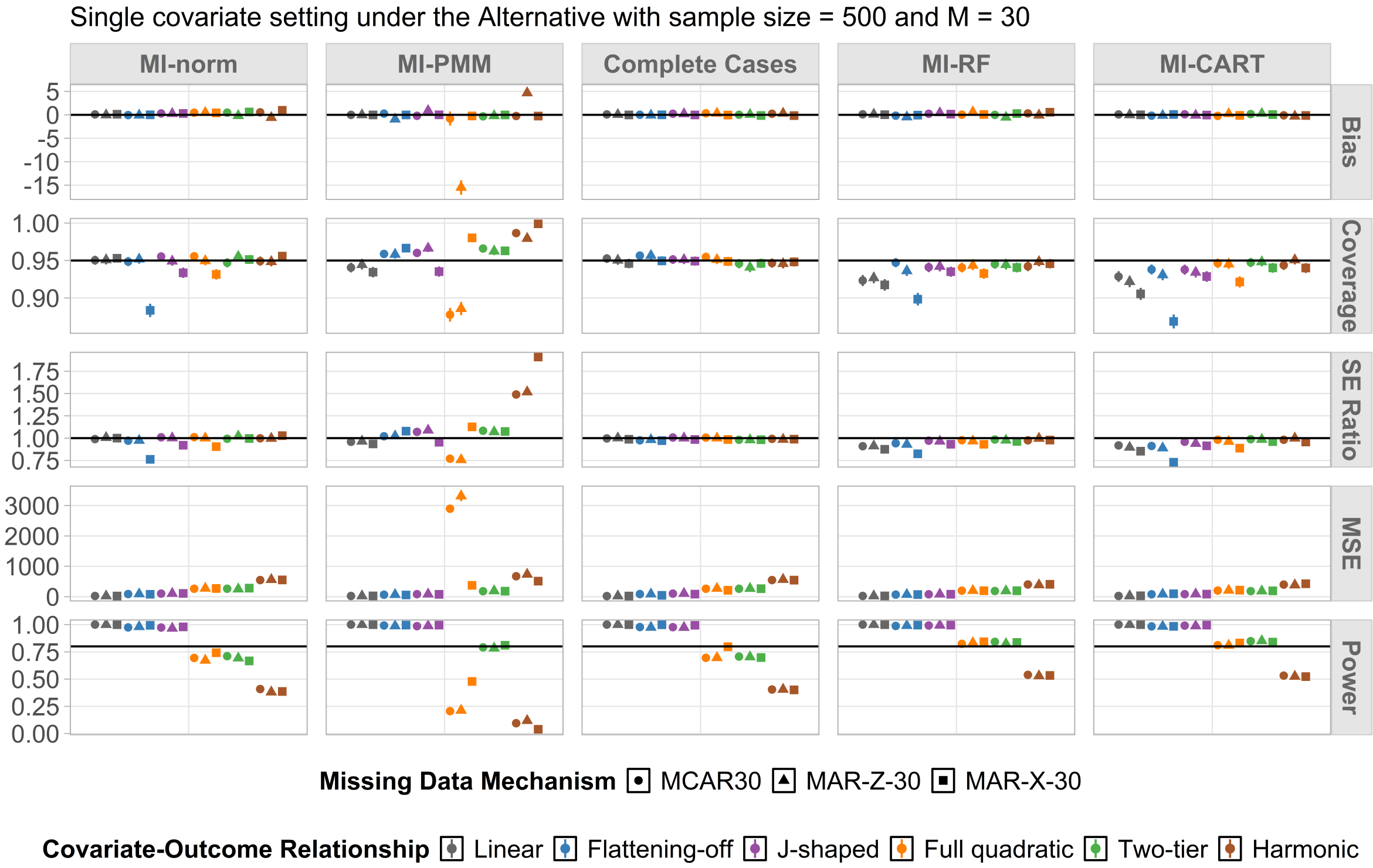

Figure 15: Simulation results for the single covariate, no interaction setting for sample size 500 when the true treatment effect is 40. The number of imputations is 30. Performance of missing data methods in terms of bias, ratio of model-based versus empirical standard error and power are provided across seven different missing data mechanisms. Note: for RF, the number of trees is set to 5 and for CART, minbucket is set to 5. Estimates are indicated with $\pm 1.96\times$ Monte Carlo error bars (but are often too small to be seen due to the scale of the plots).

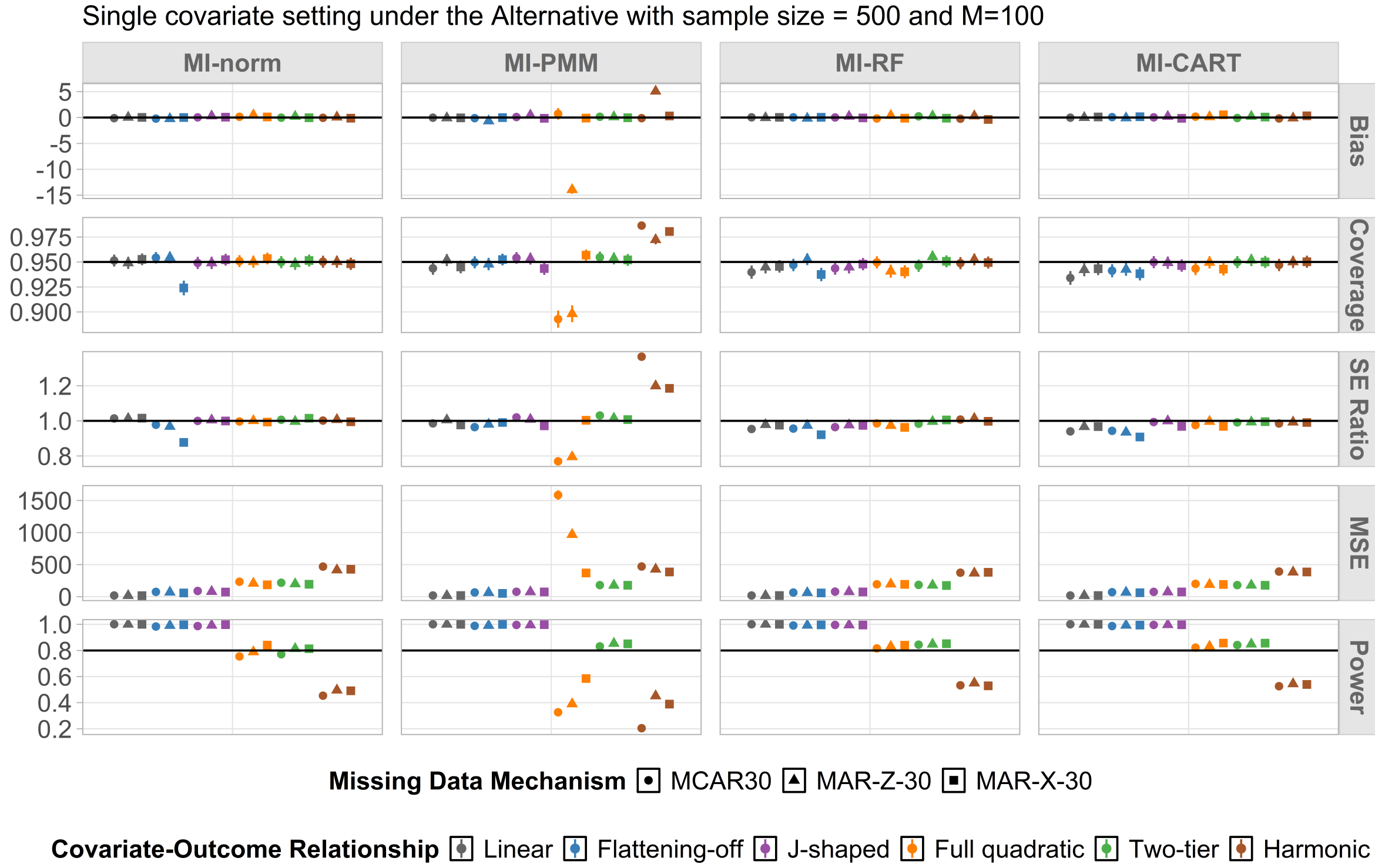

Figure 16: Simulation results for the single covariate, no interaction setting for sample size 500 when the true treatment effect is 40. The number of imputations is 100. Performance of missing data methods in terms of bias, ratio of model-based versus empirical standard error and power are provided across seven different missing data mechanisms. Note: for RF, the number of trees is set to 5 and for CART, minbucket is set to 5. Estimates are indicated with $\pm 1.96\times$ Monte Carlo error bars (but are often too small to be seen due to the scale of the plots).

2 Interaction setting

2.1 Sample size 50

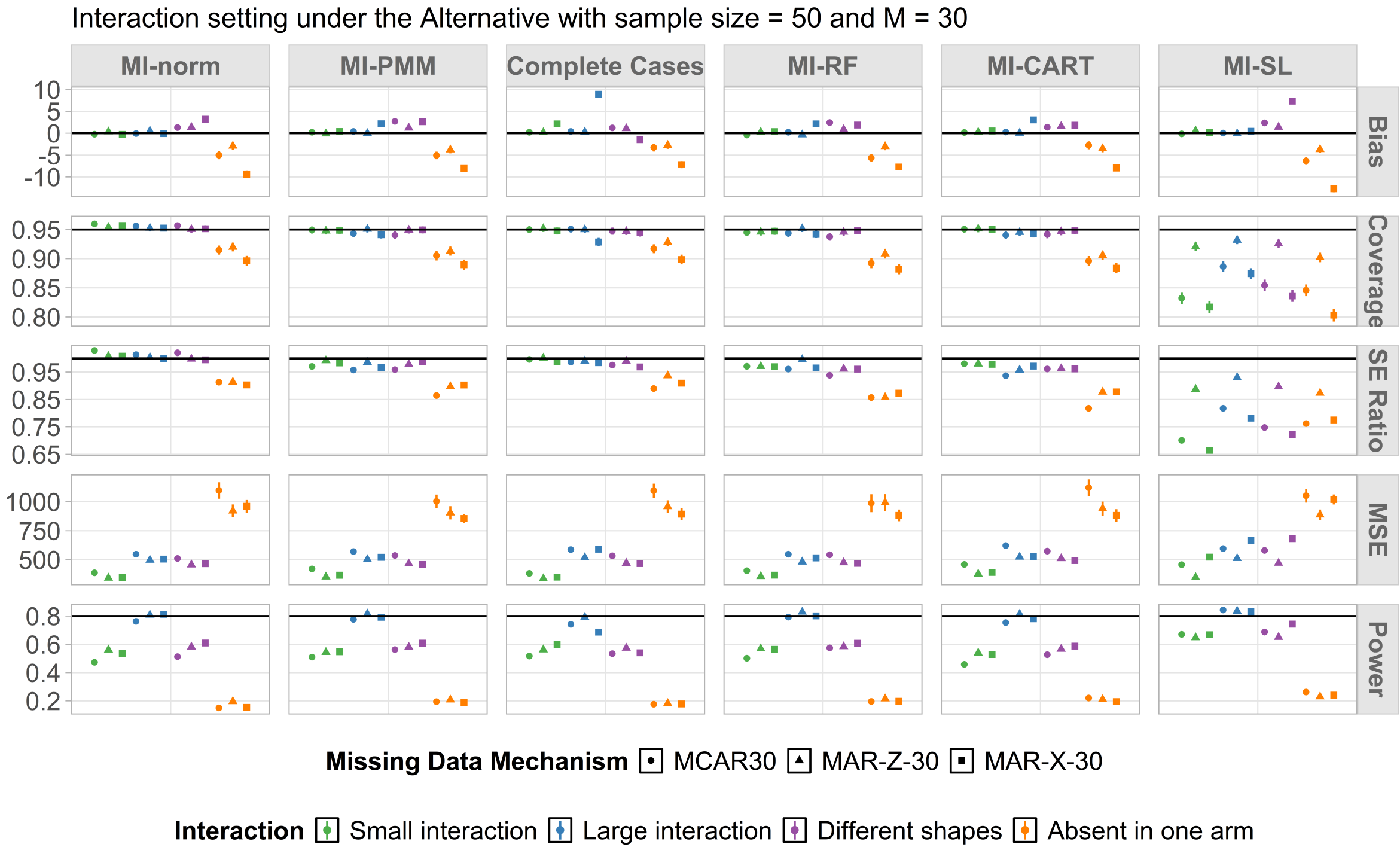

Figure 17: Simulation results for Interaction setting under the alternative when sample size is 50. Performance of missing data methods in terms of bias, coverage, ratio of model-based versus empirical standard error and power are shown. Note: for RF, the number of trees is set to 5 and for CART, minbucket is set to 5. Estimates are indicated with $\pm 1.96\times$ Monte Carlo error bars (but are often too small to be seen due to the scale of the plots).

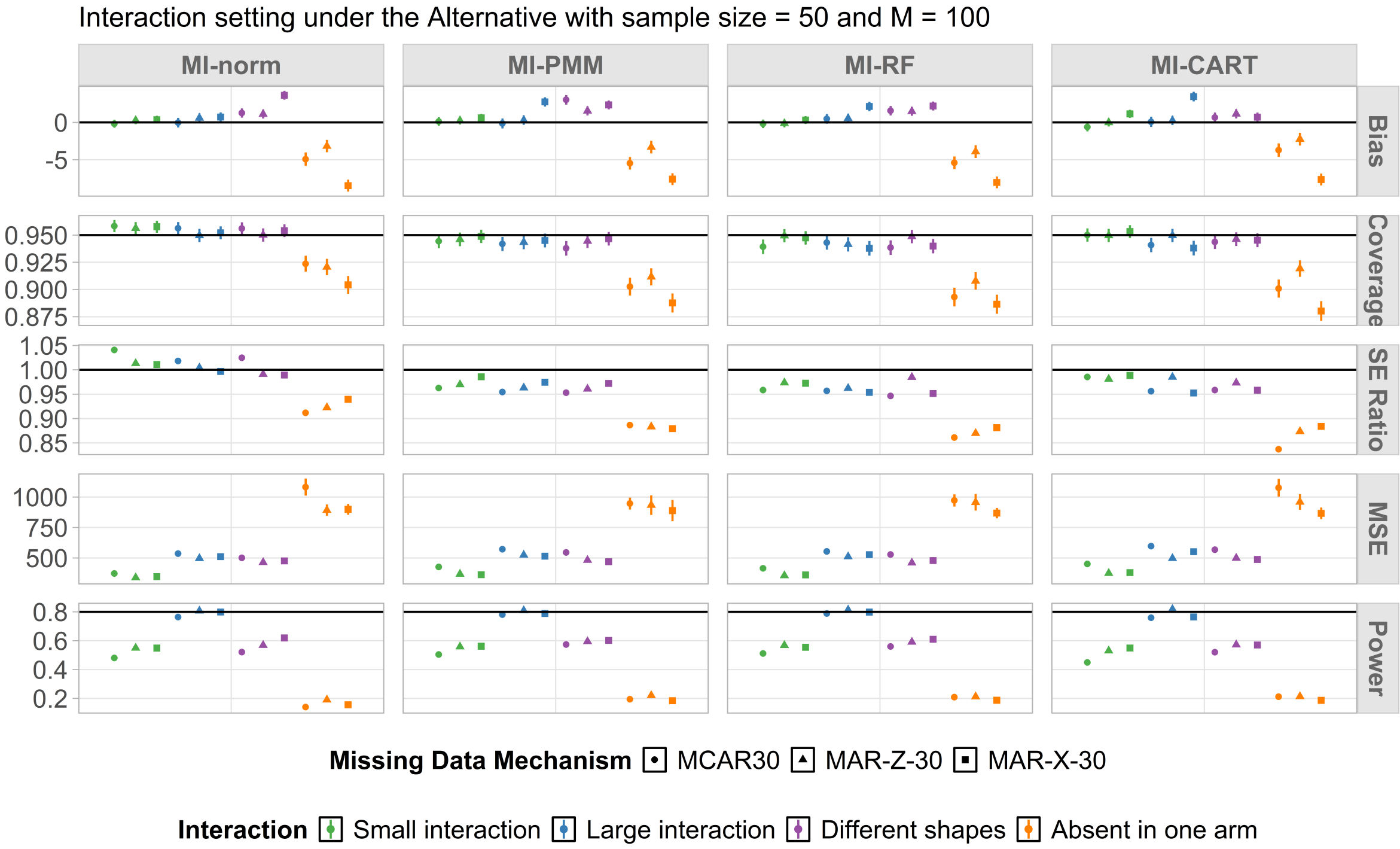

Figure 18: Simulation results for Interaction setting under the alternative when sample size is 50. Performance of missing data methods in terms of bias, coverage, ratio of model-based versus empirical standard error and power are shown. Note: for RF, the number of trees is set to 5 and for CART, minbucket is set to 5. Estimates are indicated with $\pm 1.96\times$ Monte Carlo error bars (but are often too small to be seen due to the scale of the plots).

2.2 Sample size 100

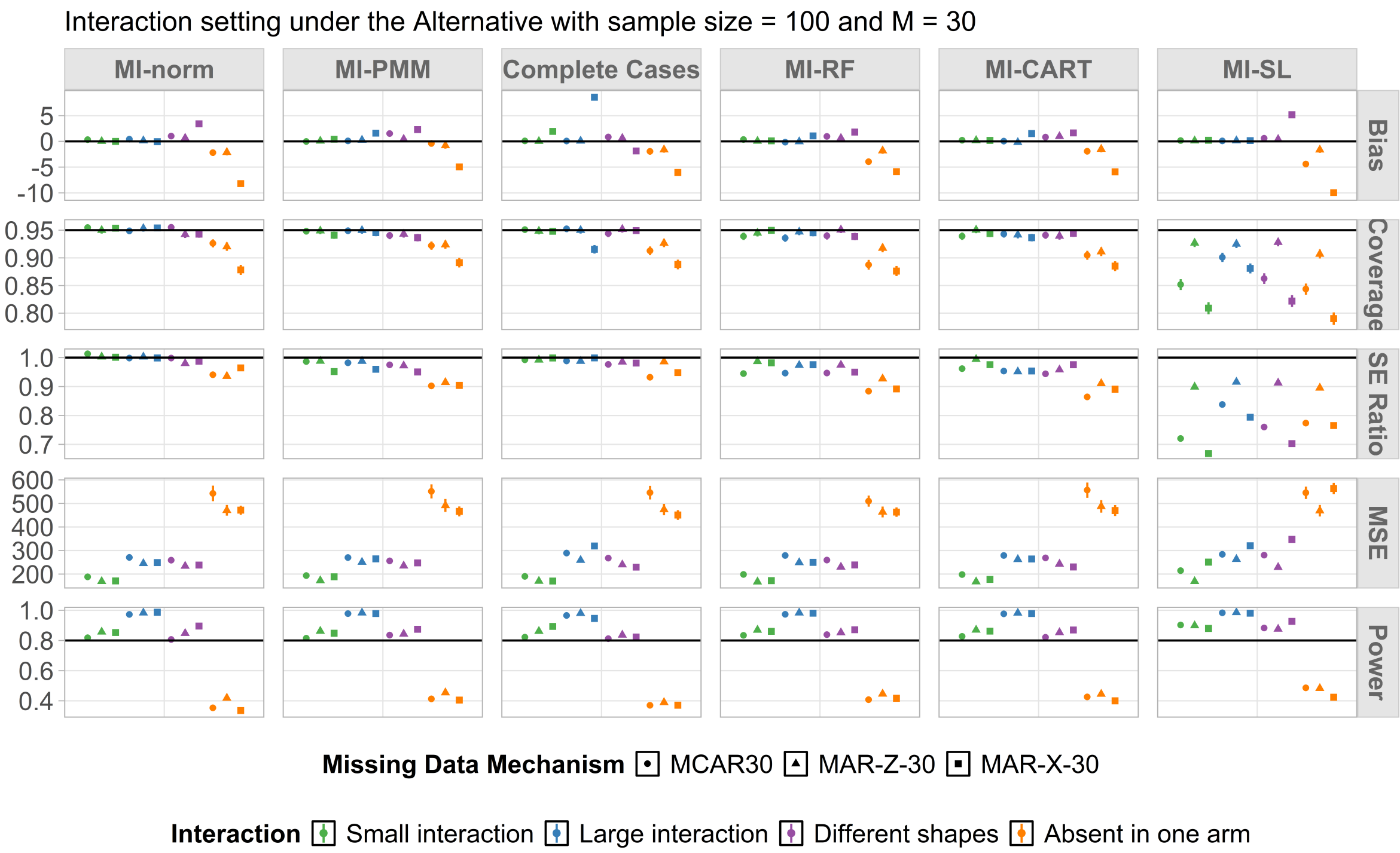

Figure 19: Simulation results for Interaction setting under the alternative when sample size is 100. The number of imputations is 30. Performance of missing data methods in terms of bias, coverage, ratio of model-based versus empirical standard error and power are shown. Note: for RF, the number of trees is set to 5 and for CART, minbucket is set to 5. Estimates are indicated with $\pm 1.96\times$ Monte Carlo error bars (but are often too small to be seen due to the scale of the plots).

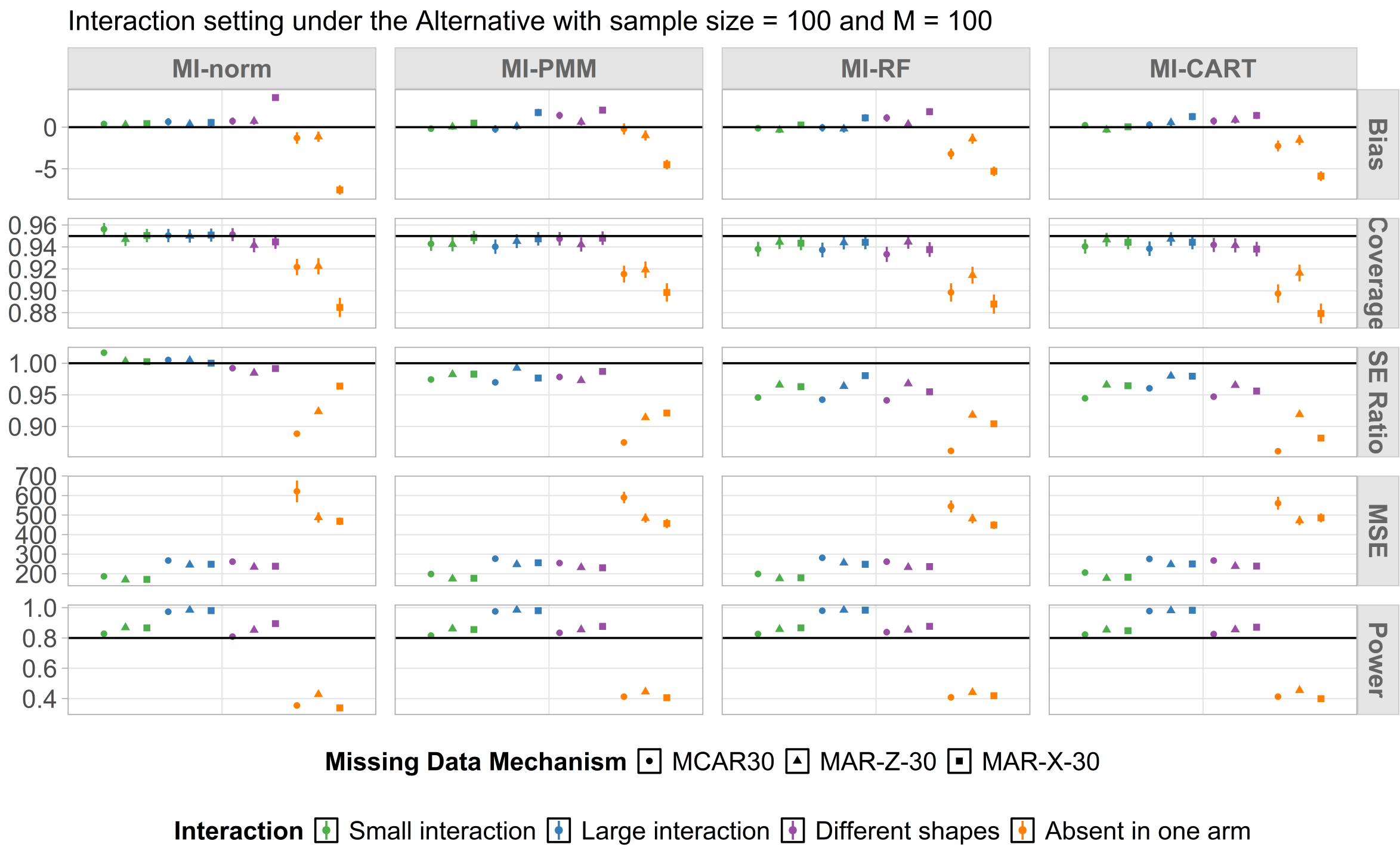

Figure 20: Simulation results for Interaction setting under the alternative when sample size is 100. The number of imputations is 100. Performance of missing data methods in terms of bias, coverage, ratio of model-based versus empirical standard error and power are shown. Note: for RF, the number of trees is set to 5 and for CART, minbucket is set to 5. Estimates are indicated with $\pm 1.96\times$ Monte Carlo error bars (but are often too small to be seen due to the scale of the plots).

2.3 Sample size 200

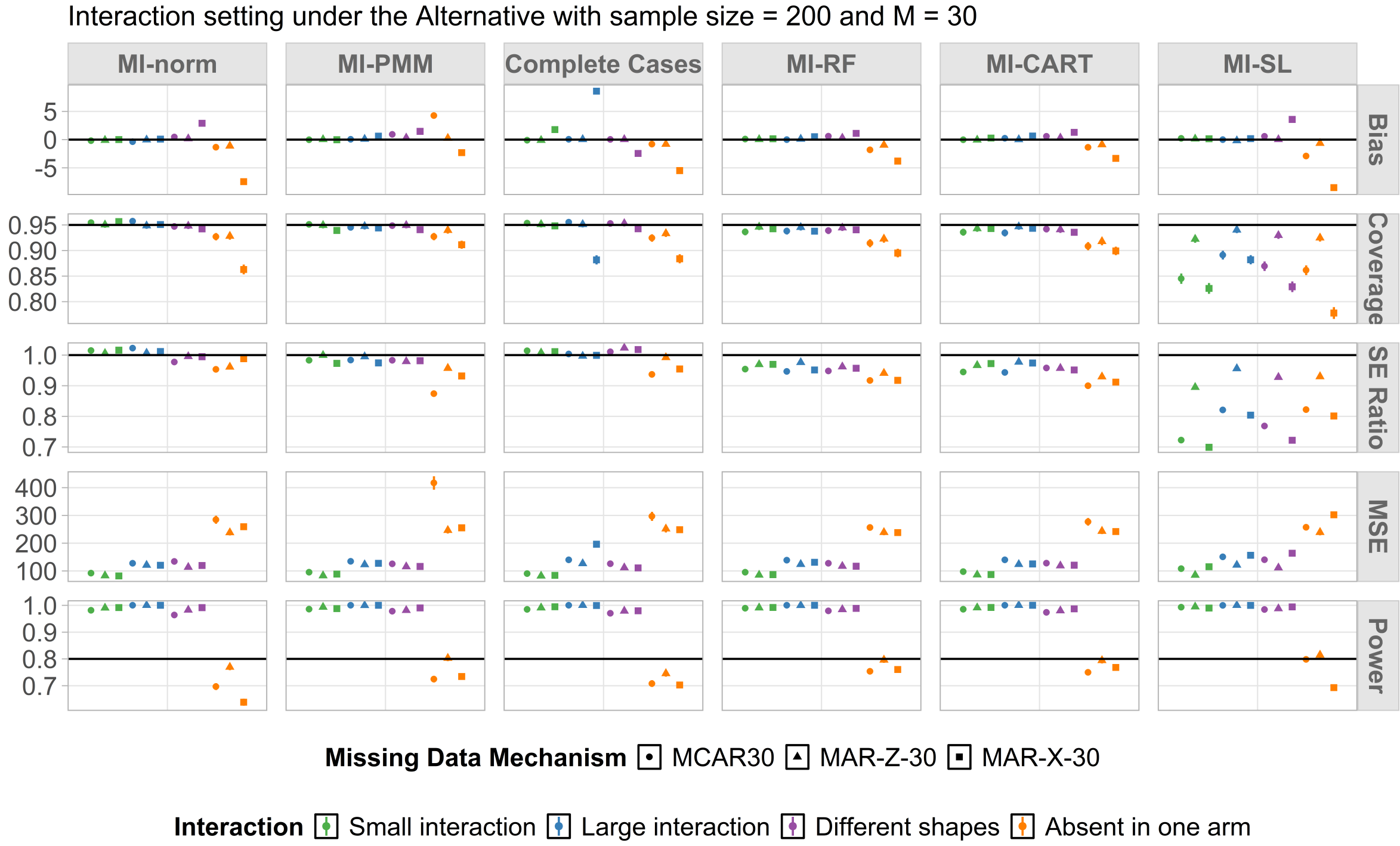

Figure 21: Simulation results for Interaction setting under the alternative when sample size is 200. The number of imputations is 30. Performance of missing data methods in terms of bias, coverage, ratio of model-based versus empirical standard error and power are shown. Note: for RF, the number of trees is set to 5 and for CART, minbucket is set to 5. Estimates are indicated with $\pm 1.96\times$ Monte Carlo error bars (but are often too small to be seen due to the scale of the plots).

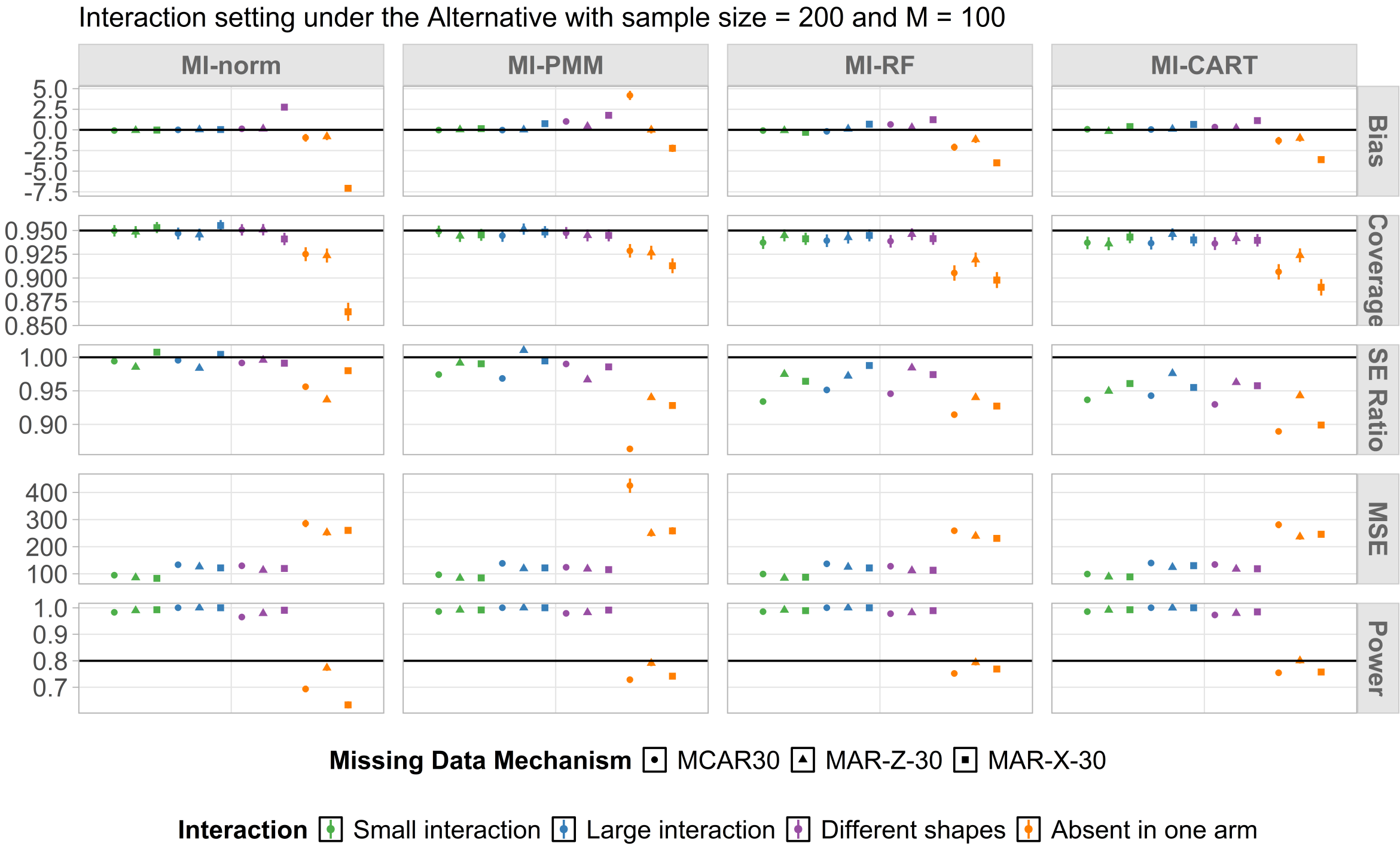

Figure 22: Simulation results for Interaction setting under the alternative when sample size is 200. The number of imputations is 100. Performance of missing data methods in terms of bias, coverage, ratio of model-based versus empirical standard error and power are shown. Note: for RF, the number of trees is set to 5 and for CART, minbucket is set to 5. Estimates are indicated with $\pm 1.96\times$ Monte Carlo error bars (but are often too small to be seen due to the scale of the plots).

2.4 Sample size 500

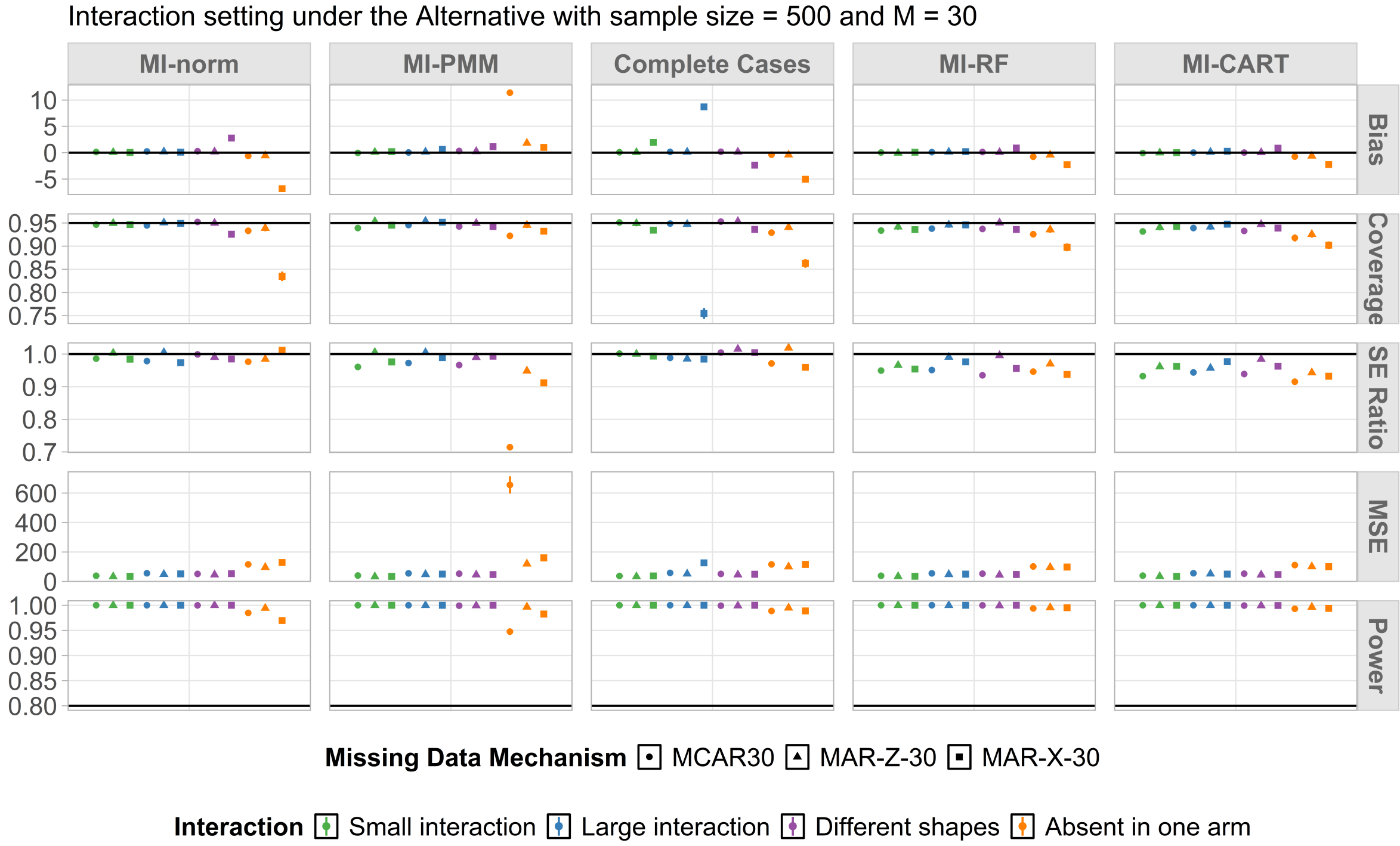

Figure 23: Simulation results for Interaction setting under the alternative when sample size is 500. The number of imputations is 30. Performance of missing data methods in terms of bias, coverage, ratio of model-based versus empirical standard error and power are shown. Note: for RF, the number of trees is set to 5 and for CART, minbucket is set to 5. Estimates are indicated with $\pm 1.96\times$ Monte Carlo error bars (but are often too small to be seen due to the scale of the plots).

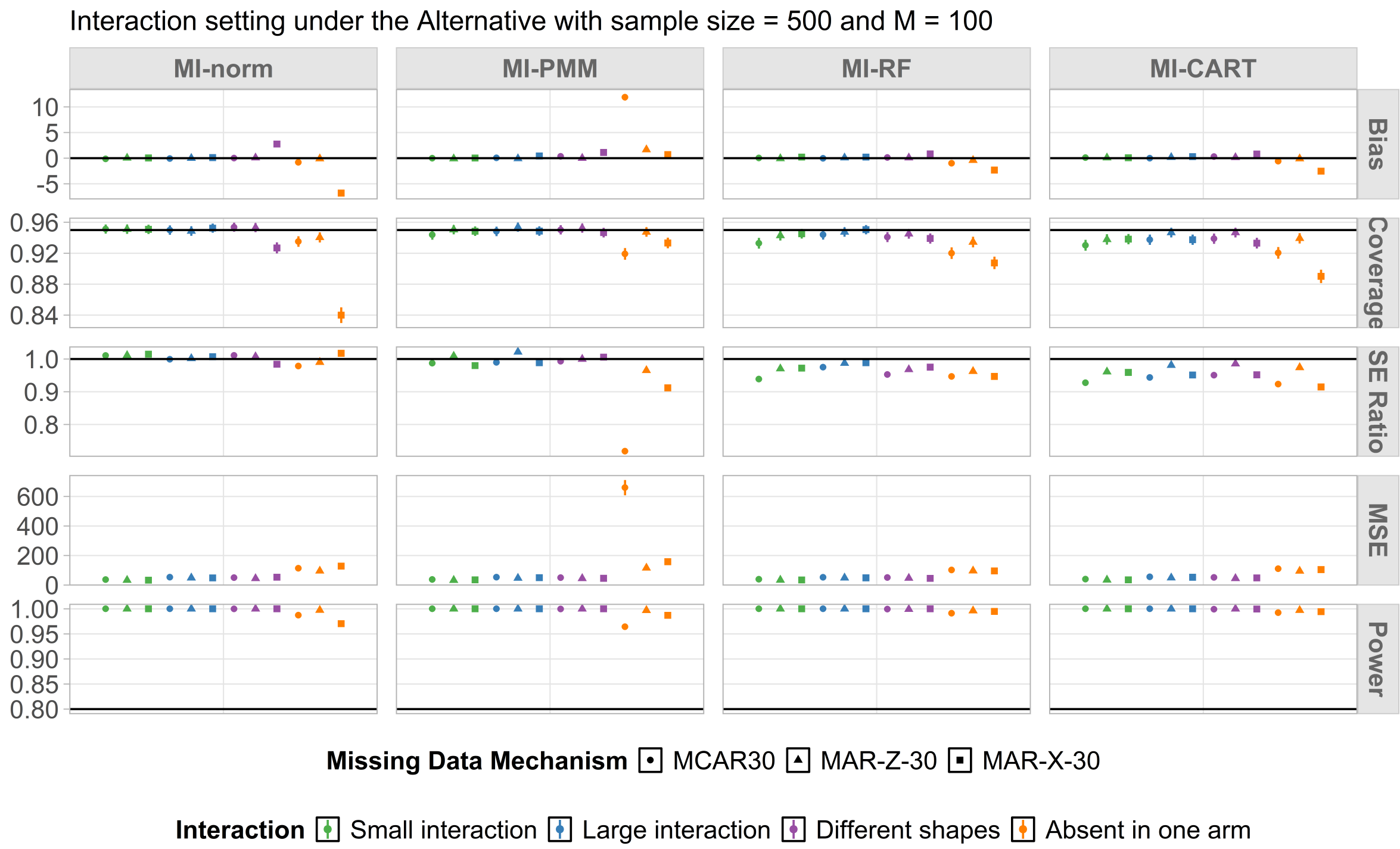

Figure 24: Simulation results for Interaction setting under the alternative when sample size is 500. The number of imputations is 100. Performance of missing data methods in terms of bias, coverage, ratio of model-based versus empirical standard error and power are shown. Note: for RF, the number of trees is set to 5 and for CART, minbucket is set to 5. Estimates are indicated with ±1.96× Monte Carlo error bars (but are often too small to be seen due to the scale of the plots).
