# Supplementary material for: Comparison of parametric versus machine-learning multiple imputation in clinical trials with missing continuous outcomes
Source: BMC Med Res Methodol. 2026 Aug 3;26:232. doi: 10.1186/s12874-026-02962-6 (PMC13721589; doi:10.1186/s12874-026-02962-6)
Supplement: Supplementary file 3 [file 12874_2026_2962_MOESM3_ESM.pdf]

Supplementary File 3

We show that, under specific missing data mechanisms (stated below), complete case analysis produces valid inference in settings explored in our first simulation study, specifically when there are (1) non-linearities in the covariate-outcome relationship and (2) treatment-covariate interactions.

1 Non-linear, no interaction simulations

In the non-linear simulations, outcomes are generated from:

$$Y_i = \beta_0 + \beta_1 Z_i + f(X_i) + \epsilon_i, \tag{1}$$

Under this model, the true *average* treatment effect is given by

$$\begin{aligned} E(Y_i|Z_i = 1) - E(Y_i|Z_i = 0) &= \beta_0 + \beta_1 + E(f(X_i)|Z_i = 1) - \{\beta_0 + E(f(X_i)|Z_i = 0)\} \\ &= \beta_1 \end{aligned}$$

since by randomisation $E(f(X_i)|Z_i = 1) = E(f(X_i)|Z_i = 0)$ because X and Z are independent.

To establish when complete case analysis yields valid inference, we derive an expression for the true average treatment effect among the complete cases ($R = 0$):

$$\begin{aligned} E(Y_i|Z_i = 1, R_i = 0) - E(Y_i|Z_i = 0, R_i = 0) = &\beta_0 + \beta_1 + E(f(X_i)|Z_i = 1, R_i = 0) - \\ &\{\beta_0 + E(f(X_i)|Z_i = 0, R_i = 0)\}. \end{aligned}$$

This will equal β_1 (the true average treatment effect in the full data) whenever $E(f(X_i)|Z_i = 1, R_i = 0) = E(f(X_i)|Z_i = 0, R_i = 0)$. This holds when outcomes are missing completely at random, since then R is independent of (X, Z), and so

$$\begin{aligned} E(f(X_i)|Z_i = 1, R_i = 0) &= E(f(X_i)|Z_i = 1) \\ &= E(f(X_i)|Z_i = 0) \\ &= E(f(X_i)|Z_i = 0, R_i = 0). \end{aligned}$$

It also holds when missingness depends only on treatment Z, since then X is independent of

(Z, R) and so

$$E(f(X_i)|Z_i = 1, R_i = 0) = E(f(X_i))$$
$$= E(f(X_i)|Z_i = 0, R_i = 0).$$

Lastly, it holds when missingness depends only on covariate X, since then treatment Z is independent of (X, R), and so

$$E(f(X_i)|Z_i = 1, R_i = 0) = E(f(X_i)|R_i = 0)$$
$$= E(f(X_i)|Z_i = 0, R_i = 0).$$

Thus in the three missingness mechanism types used in our simulations, the true complete case average treatment effect equals β_1, the true average treatment effect in the full data. As such, the results of [Wang et al., 2019] imply that the complete case treatment effect estimator is unbiased despite the analysis model being misspecified.

The results of [Wang et al., 2019] also show that the ANCOVA standard error is consistent for the true standard error, but this is under an assumption of 1:1 randomisation. While in our simulations randomisation was 1:1, among the complete cases treatment groups are balanced (in expectation) in the MCAR and MAR-X missingness scenarios, but not in the MAR-Z missingness scenario.

In the MAR-Z case, where there will be an imbalance in the number of patients in each treatment group among the complete cases, the results of [Bartlett, 2020] imply that the usual ANCOVA standard error estimator is still consistent if $E(Y|Z, X, R = 0)$ is linear in Z and some function of X and $Var(Y|Z = 0, X, R = 0) = Var(Y|Z = 1, X, R = 0)$. These conditions hold in the full data (i.e. without conditioning on $R = 0$) under our no-interaction data generating mechanism, and so also hold under treatment dependent missingness.

In conclusion, the complete case ANCOVA treatment effect estimator and its corresponding standard error are both consistent under the no-interaction non-linear simulations and under the three types of missingness mechanism considered.

2 Interaction simulations

We now consider when complete case analysis is valid for the interaction simulations. To do so, we write

$$Y_i = \beta_0 + g_0(X_i) + Z_i g_1(X_i) + \epsilon_i,$$

to denote the data generating mechanism, where $g_0(X_i)$ and $g_1(X_i)$ are two specified functions. The full data true average treatment effect is then

$$\begin{aligned}E(Y_i|Z_i=1)-E(Y_i|Z_i=0)=&\beta_0+E(g_0(X_i)|Z_i=1)+E(g_1(X_i)|Z_i=1)-\\&\{\beta_0+E(g_0(X_i)|Z_i=0)\}\\=&E(g_1(X_i))\end{aligned}$$

using the fact that by randomisation, X and Z are independent.

The complete case true average treatment effect is then

$$\begin{aligned}E(Y_i|Z_i=1,R_i=0)-E(Y_i|Z_i=0,R_i=0)=&\beta_0+E(g_0(X_i)|Z_i=1,R_i=0)\\&+E(g_1(X_i)|Z_i=1,R_i=0)\\&-\{\beta_0+E(g_0(X_i)|Z_i=0,R_i=0)\}\\=&E(g_0(X_i)|Z_i=1,R_i=0)\\&+E(g_1(X_i)|Z_i=1,R_i=0)\\&-E(g_0(X_i)|Z_i=0,R_i=0).\end{aligned}$$

Under MCAR, R is independent of (X,Z). This means

$$\begin{aligned}E(g_0(X_i)|Z_i=1,R_i=0)&=E(g_0(X_i))\\&=E(g_0(X_i)|Z_i=0,R_i=0)\end{aligned}$$

and $E(g_1(X_i)|Z_i=1,R_i=0)=E(g_1(X_i))$, and so the complete case true average treatment effect equals $E(g_1(X_i))$, i.e. the same as the full data true average treatment effect.

Under MAR-Z, X is independent of (Z,R), and so

$$\begin{aligned}E(g_0(X_i)|Z_i=1,R_i=0)&=E(g_0(X_i))\\&=E(g_0(X_i)|Z_i=0,R_i=0)\end{aligned}$$

and

$$E(g_1(X_i)|Z_i=1,R_i=0)=E(g_1(X_i))$$

and so again the complete case true average treatment effect equals the full data true average treatment effect.

Lastly, under MAR-X, Z is independent of (X,R), and so

$$\begin{aligned}E(g_0(X_i)|Z_i=1,R_i=0)&=E(g_0(X_i)|R_i=0)\\&=E(g_0(X_i)|Z_i=0,R_i=0),\end{aligned}$$

such that these terms again cancel out. However,

$$
\begin{aligned}
E(g_1(X_i)|Z_i = 1, R_i = 0) &= E(g_1(X_i)|R_i = 0) \\
&\neq E(g_1(X_i))
\end{aligned}
$$

and so the complete case true average treatment effect differs to the full data effect. Thus the complete case ANCOVA estimator is unbiased under MCAR and MAR-Z, but not under MAR-X missingness.

As in the non-linear simulations, under MCAR the results of [Wang et al., 2019] demonstrate the standard error estimator will be consistent. Under MAR-Z, as in the non-linear simulations, their results do not apply due to the fact that the numbers of patients in the two arms are not balanced (in expectation) among the complete cases. Moreover, under imbalance, the results from [Bartlett, 2020] indicate the standard error estimator will not be consistent under the interaction data generating model in general. In conclusion, the theoretical results suggest the complete case standard errors are only consistent under MCAR. We note however that this could be remedied by use of a sandwich standard error.

References

[Bartlett, 2020] Bartlett, J. W. (2020). Robustness of ancova in randomized trials with unequal randomization. *Biometrics*, 76(3):1036–1038.

[Wang et al., 2019] Wang, B., Ogburn, E. L., and Rosenblum, M. (2019). Analysis of covariance in randomized trials: More precision and valid confidence intervals, without model assumptions. *Biometrics*, 75(4):1391–1400.
